# A hierarchical approach for evaluating athlete performance with an application in elite basketball

**DOI:** 10.1038/s41598-024-51232-2

**Published:** 2024-01-19

**Authors:** Thiago de Paula Oliveira, John Newell

**Affiliations:** 1 School of Mathematical and Statistical Sciences, University of Galway, Galway, Ireland; 2 The Insight Centre for Data Analytics, University of Galway, Galway, Ireland; 3 Orreco Ltd, Galway, Ireland

**Keywords:** Statistics, Applied mathematics

## Abstract

In this paper, we present the ON score for evaluating the performance of athletes and teams that includes a season-long evaluation system, a single-game evaluation, and an evaluation of an athlete’s overall contribution to their team. The approach used to calculate the ON score is based on mixed-effects regression models that take into account the hierarchical structure of the data and a principal component analysis to calculate athlete rating. We apply our methodology to a large dataset of National Basketball Association (NBA) games spanning four seasons from 2015–2016 to 2018–2019. Our model is validated using two systematic approaches, and our results demonstrate the reliability of our approach to calculate an athlete’s performance. This provides coaches, General Managers and player agents with a powerful tool to gain deeper insights into their players’ performance, make more informed decisions and ultimately improve team performance. Our methodology has several key advantages. First, by incorporating the hierarchical structure of the data, we can obtain valuable information about an athlete’s contribution within their team. Second, the use of principal component analysis allows us to calculate a single score, the ON score, that captures the overall performance of an athlete. Third, our approach is based on classical restricted likelihood methods, which makes the calculation faster than Bayesian methods typically requiring 1000 posterior samples. With our approach, coaches and managers can evaluate athletes’ performance throughout the season, compare athletes and teams over a year, and assess an athlete’s performance during a single game. Our methodology can also complement other ratings and box score metrics to provide a more comprehensive assessment of an athlete’s performance as our method uses the hierarchical nature of performance data (i.e. player nested within team over season) which is typically ignored in player rating systems. In summary, our methodology represents a significant contribution to the field of sports analytics and provides the foundation for future developments.

## Introduction

Over the past decade, the employment of sophisticated statistical methods in sport has grown increasingly significant for evaluating the performance of teams and athletes^1–3^. These methods are most prevalent in basketball, ice hockey, and football, where the objective is to create rating systems that gauge the quality of athletes and teams^3^. Although team ratings have been established, devising individual ratings for athletes within a team remains challenging. As a result, pinpointing and quantifying each athlete’s individual contribution during a given season is vital for clubs, coaches, and managers to identify and recruit talented, undervalued athletes. According to Hass and Craig^4^, ratings can also be useful for sports enthusiasts wishing to evaluate the performance of their favourite athletes or teams. Moreover, these ratings can be applied to video games, as demonstrated by research from Baayen et al.^5^ and Matano et al.^6^. Specifically, rating systems in video games can be utilised to assess the skills of virtual athletes and enhance the overall gaming experience.

In basketball for example, various factors, such as shot attempts, assists, rebounds, turnovers, blocked shots, fouls, and others, have been employed to establish criteria for evaluating athletes^3,7,8^. However, in order to avoid subjective assessments, which are often biased, the development of statistical learning algorithms is necessary^9^. Regrettably, a considerable number of research studies on athlete ratings have been published without peer review, as noted by Hvattum^3^, with some even described in internet blog posts, such as the Athlete Efficiency Rating (PER). According to these authors, Bayesian or likelihood-based models, as well as logistic, ridge, or lasso regression, are the most commonly used statistical methods for calculating athlete ratings, with some employing simple calculations. Although some of these approaches incorporate advanced statistical methods for calculating ratings^10–13^, no method has yet been developed to account for hierarchical structures in the calculation of athlete ratings.

In this context, incorporating a natural variance-covariance structure can offer specific insights into the impact of athletes within a team by season, enabling clubs and coaches to more effectively assess the technical and tactical efficiency of their athletes or compare them across matches or seasons^12,14^. Moreover, the creation of less subjective athlete evaluations can prove beneficial for club management, athletes, fans, games, and the media. Alongside the aforementioned variables, other aspects such as player positions, roles, and team strategies ought to be taken into account when determining athlete ratings. For instance, point guards in basketball may be assigned a higher rating if they excel in assists and ball handling, while centres might receive a higher rating if they are proficient in rebounds and blocked shots. Furthermore, an athlete’s performance impact on their team’s overall success should be considered when calculating ratings.

Utilising mixed-effects regression models can present a promising alternative for assessing the performance of athletes and teams throughout a season. When multiple outcomes or covariates are repeatedly measured within a group of individuals, randomly chosen from one or more populations, a multilevel model can shed light on how each athlete contributes to their team during a season^15–17^. For example, when developing a new rating to gauge an athlete’s contribution across multiple games using *n* explanatory variables in the linear predictor, a multilevel model can help ascertain how each athlete contributes to their team over the course of a season^18^. Multilevel models boast several advantages over traditional statistical methods, such as employing Restricted Maximum Likelihood (REML) to obtain more accurate estimates of variance components and the ability to handle unbalanced data and missing data^15,19^. Furthermore, multilevel models can offer higher statistical power in the presence of missing data, reduce the standard errors of the estimators and can accommodate different forms of random effects^17^.

In this paper, we introduce a novel method for assessing the performance of athletes and teams, specifically focusing on an athlete’s contribution to their team throughout a season, a metric for comparing athletes and teams across a season, and an evaluation for examining athletes during individual matches. Our approach utilises principal components to determine an athlete’s rating and employs mixed-effects regression models to accommodate the hierarchical structure of the data. While our initial application centres on basketball data, the methodology can be readily extended to other sports.

## Example of application in the NBA data

**Figure 1:**
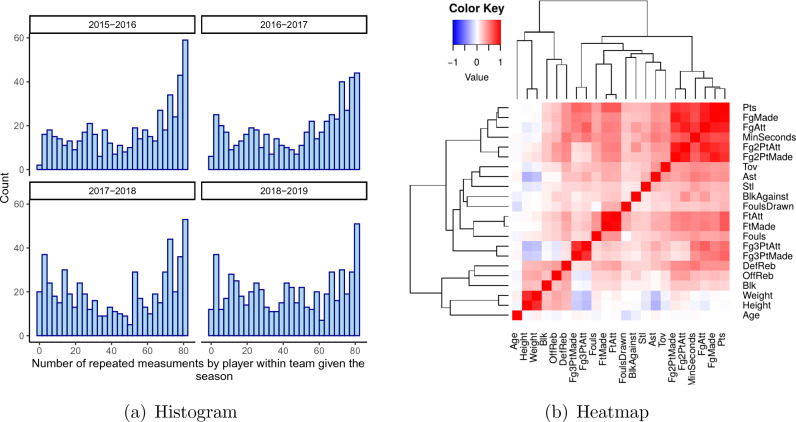
(**a**) Histogram displaying athlete game participation across 26 NBA Teams for each season, and (**b**) Heatmap of Pearson correlation between 22 pairs of performance variables with hierarchical clustering.

In this section, we apply the proposed methodology to a large dataset of NBA games covering the 2015–2016 to 2018–2019 seasons, i.e. four NBA seasons in total. In a typical season, each of the 30 teams plays 82 games, resulting in a total of 1230 regular-season games played during the season. With four seasons in our dataset, we have 4920 regular games and 900 athletes available in the database resulting in a varying number of repeated measurements per athlete within a team in a season, as shown in Fig. 1a. The number of repeated measurements can vary between 1 and 82 during the season, which makes it difficult to evaluate the performance of athletes when they only play a few games, as we do not have enough information about their performance during the season. A low number of repeated measures could occur when an athlete is affected by injury, as discussed by Deitch et al.^20^.

Figure 1b shows a heatmap showing the Pearson correlation coefficient between each pair of variables together with hierarchical clustering based on Ward’s hierarchical agglomerative clustering method^21,22^. The heatmap shows two main groups of variables: The first group includes physical, block and rebound information, while the second group includes offensive and defensive metrics. The variables generally show moderate negative or positive correlation with each other, with some high correlations (as expected) between height and weight and between attempted and scored points.

Table 1 shows the sample mean, standard deviation, coefficient of variation and PCA-based weight $({\textbf{W}})$ for the 22 response variables. It is interesting to note that the athletes’ height has a low coefficient of variation, indicating almost complete equality between the athletes, while the number of fouls drawn during the game has a high coefficient of variation, indicating that the athletes vary in their ability to draw fouls during the game. When we multiply the scaled response variables by their respective coefficient of variation, all with the highest coefficient of variation have high control over the influence of the early principal components, but do not necessarily affect the ON score, in the same way, as shown by the PCA-based weight in Table 1. Therefore, it is important to consider both the coefficient of variation and the PCA-based weight to understand the influence of each response variable on the ON score.

**Table 1:** Sample mean, standard deviation (SD), coefficient of variation (CV) and estimated PCA-based weight ($\widehat{{\textbf{W}}}$) used to calculate the ON for each response variable.

Response	Mean	SD	CV	$\widehat{{\textbf{W}}}$	Response	Mean	SD	CV	$\widehat{{\textbf{W}}}$
Height	200.82	8.72	0.04	− 0.0095	Off reb.	0.96	1.35	1.41	0.6155
Weight	99.81	11.40	0.11	0.0556	Def. reb.	3.20	2.75	0.86	0.3802
Age	28.73	4.46	0.16	− 0.1587	Ast	2.19	2.48	1.13	1.9438
Fg2Pt att.	5.51	4.44	0.81	1.6182	Pts	10.06	8.12	0.81	1.7776
Fg2Pt made	2.79	2.59	0.93	1.8569	Tov	1.28	1.39	1.08	0.3196
Fg3Pt att.	2.65	2.71	1.02	0.6787	Stl	0.73	0.97	1.34	0.4579
Fg3Pt made	0.95	1.32	1.39	1.8344	Blk	0.46	0.84	1.82	− 0.4559
Fg att.	8.15	5.64	0.69	1.3132	Blk against	0.46	0.76	1.64	− 1.5967
Fg made	3.73	3.03	0.81	1.8512	Min. seconds	1366.42	620.54	0.45	0.4390
Ft att.	2.15	2.77	1.28	0.9371	Fouls	2.65	2.41	0.91	1.5422
Ft made	1.65	2.27	1.38	1.4119	Fouls drawn	0.50	1.34	2.70	0.3843

An important aspect of our methodology, as indicated in Table 1, is the flexibility it offers practitioners in selecting input variables. While each variable uniquely influences the model, guided by its coefficient of variation and PCA-based weight, practitioners have the discretion to determine which variables are most relevant for their specific context. This is not a weakness of the method but rather an advantage, empowering users to tailor the model to their needs. This adaptability highlights the model’s applicability across different sports analytic scenarios. However, it is important to understand that altering input variables can affect the model’s covariance structure and PCA weights, and thus the stability of the ON score.

After calculating the ON score for each player in each game, we included it as a new response variable in a multilevel model containing the corresponding hierarchical structure and main and interaction effects such as rookie, position and game location. The aim was to identify the systematic effects of these variables on the ON score. To this end, we fitted the model (3) adding all first-order interaction effects and followed the model selection procedure outlined in the Methods section. The results of the analysis of variance type III using the Satterthwaite method to calculate the correct degrees of freedom and the corresponding *p*-values of the F-test statistics for the main effects and interactions can be found in Table 2.

**Table 2:** Type III analysis of variance table with Satterthwaite’s method.

Source of variation	Sum sq.	Mean sq.	Num. DF	Den. DF	F value	Pr (> F)
Venue	0.0163	0.0163	1	101802.28	112.936	< 0.0001
Position	0.0554	0.0092	6	2569.76	63.948	< 0.0001
Rookie	0.0075	0.0075	1	2286.54	51.658	< 0.0001
poly(Pts, 3)	26.8573	8.9524	3	103315.16	61976.612	< 0.0001
poly(Blk against, 2)	4.8755	2.4377	2	102407.81	16876.060	< 0.0001
poly(Ast, 3)	11.4443	3.8148	3	103364.51	26409.192	< 0.0001
poly(Ft att, 2)	0.7208	0.3604	2	102581.40	2494.899	< 0.0001
poly(Off reb, 2)	3.5147	1.7573	2	102756.52	12165.880	< 0.0001
Interactions	6.2316	0.0878	71	102487.02	607.618	< 0.0001

We ran a mixed-effects ANCOVA model to examine the factors explaining variation in the ON score. The full model included all first-level interaction effects between the explanatory variables. A comprehensive list of all covariates considered, including those used in the initial stages of model development for the ON score, a summary of the model parameters containing estimates, standard errors, and t-tests based on Satterthwaite’s method can be found in the “Supplementary Material”. The analysis, as detailed in the full model, revealed significant two-way interaction effects. These findings provide evidence that the ON score is influenced by a complex interplay of factors. Specifically, interactions among various player characteristics and game-related variables contribute uniquely to the ON score. This highlights the intricate and multi-factorial nature of determining player performance in elite basketball, as captured by the ON score.

The variance component estimates were ${\widehat{\sigma }}_{1}=0.005136$ for the season, ${\widehat{\sigma }}_{23}=0.007496$ for the athlete within the team within the season, and ${\widehat{\sigma }} = 0.012019$ for the error term. Because the estimate of $\sigma _{2}$ was close to zero, we fitted a reduced nested model that included only one variance component to explain within-season athlete variability (${\widehat{\sigma }}_{23}$). We then tested the hypothesis $H_{0}: \sigma _{2}^2=0$ versus $H_{a}: \sigma _{2}^2 > 0$ using a likelihood ratio test based on a mixture of two $\chi ^2$ distributions. The results of the test showed strong evidence that there is no exclusive variance component term for the team (*p*-value $\approx 1$).

We calculated the intraclass correlation coefficient (ICC) to determine the proportion of variability in the ON score that could be attributed to the different levels of the hierarchy. We found that season explained approximately $\text{ ICC}_1=30.41\%$ of the total variability in the data, while season and athlete within team explained $\text{ ICC}_3=51.24\%$ in a season. This suggests that there is a high level of variation between athletes within a team within a season explained by the model.

In addition, the ON score can be adjusted for different combinations of venue, position and rookie. Combined with random effects prediction (BLUPs), this allows for specific evaluations of athletes over the course of the season per team or between teams, as well as comparisons of teams, athletes or athletes within a team during the season. In this sense, we can use the median and interquartile range of conditional means, as defined in the Methods section, as a way to rank athletes based on point and interval estimates. Table 3 shows the top 10 athletes for the 2017–2018 and 2018–2019 seasons, where we can see that James Harden, Russell Westbrook and LeBron James occupy the first, second and third positions, respectively, for both seasons based on the point estimate of $\gamma ^{\tiny \text{(R) }}_{ijk}$.

**Table 3:** Top 10 athletes of seasons 2017–2018 and 2018–2019 based on relevant score $\left( {\widetilde{\gamma }}^{\tiny \text{(R) }}_{ijk}\right)$ and it 95% confidence interval, with addition of min–max scaled $\left( {\widetilde{\gamma }}^{*\tiny \text{(R) }}_{ijk}\right)$ version of ${\widetilde{\gamma }}^{\tiny \text{(R) }}_{ijk}$, and the number of games (NG) played.

Rank	Season	Athlete	${\widetilde{\gamma }}^{\tiny \text{(R) }}$	Lower	Upper	${\widetilde{\gamma }}^{*\tiny \text{(R) }}$	NG
1	2017–2018	James Harden	1.2050	1.1970	1.2153	77.71	72
2	2017–2018	Russell Westbrook	1.2014	1.1906	1.2086	77.13	80
3	2017–2018	LeBron James	1.1979	1.1837	1.2026	76.57	82
4	2017–2018	Stephen Curry	1.1874	1.1729	1.1965	74.90	51
5	2017–2018	Giannis Antetokounmpo	1.1863	1.1716	1.1917	74.72	75
6	2017–2018	Damian Lillard	1.1847	1.1734	1.1909	74.47	73
7	2017–2018	Anthony Davis	1.1826	1.1660	1.1895	74.13	75
8	2017–2018	DeMarcus Cousins	1.1773	1.1685	1.1920	73.28	48
9	2017–2018	Devin Booker	1.1724	1.1554	1.1831	72.49	54
10	2017–2018	Kevin Durant	1.1714	1.1616	1.1802	72.33	68
1	2018–2019	James Harden	1.2361	1.2226	1.2441	82.69	78
2	2018–2019	Russell Westbrook	1.1988	1.1858	1.2037	76.72	73
3	2018–2019	LeBron James	1.1956	1.1867	1.2062	76.20	55
4	2018–2019	Blake Griffin	1.1919	1.1728	1.1953	75.61	75
5	2018–2019	Paul George	1.1901	1.1814	1.2007	75.32	77
6	2018–2019	Joel Embiid	1.1874	1.1758	1.2001	74.90	64
7	2018–2019	Giannis Antetokounmpo	1.1870	1.1784	1.1996	74.82	72
8	2018–2019	Devin Booker	1.1865	1.1740	1.1997	74.75	64
9	2018–2019	Kawhi Leonard	1.1848	1.1683	1.1891	74.48	60
10	2018–2019	Anthony Davis	1.1812	1.1624	1.1911	73.90	56

The analysis of the 95% confidence interval reveals interesting findings about the athlete’s relevance. For instance, James Harden did not differ significantly from Russell Westbrook and LeBron James in 2017–2018; however, in 2018–2019, he had a significant difference from them, becoming the most relevant athlete. The confidence interval for ${\widetilde{\gamma }}^{\tiny \text{(R) }}$ of James Harden between both seasons did not overlap, indicating that Harden had a significant improvement in relevance in 2018–2019 compared to 2017–2018. It is worth noting that just classifying athletes based on their point estimates can induce classification bias. Hence, to avoid such bias, we recommend using both point and interval estimates when comparing athletes.

To further improve the evaluation of athletes, we can combine the results of the relevance score $\left( {\widetilde{\gamma }}^{\tiny \text{(R) }}\right)$ with the consistency score $\left( \widetilde{\varvec{\gamma }}_{athlete}^{\tiny \text{(CS) }}\right)$. The consistency score measures whether an athlete’s in-season performance was below, close to or above the expected performance within the team, as shown in Table 4. Combining both scores can provide a more complete and accurate assessment of an athlete’s performance, especially when comparing athletes.

**Table 4:** Ratings of the top 10 athletes by season who performed better than their expected performance within the team, with the addition of 95 per cent confidence intervals for $\varvec{\gamma }_{athlete}^{\tiny \text{(CS) }}$ generated using 10,000 Monte Carlo simulations.

Rank	Season	Team	athlete	Pos.	$\widetilde{\varvec{\gamma }}_{athlete}^{\tiny \text{(CS) }}$	Lower	Upper
1	2017–2018	Pelicans	DeMarcus Cousins	C	0.0273	0.0211	0.0336
2	2017–2018	Nuggets	Nikola Jokic	C	0.0198	0.0160	0.0236
3	2017–2018	Grizzlies	Marc Gasol	C	0.0192	0.0101	0.0284
4	2017–2018	Thunder	Paul George	SF	0.0189	0.0161	0.0216
5	2017–2018	76ers	Joel Embiid	C	0.0182	0.0119	0.0245
6	2017–2018	Pistons	Blake Griffin	PF	0.0177	0.0149	0.0205
7	2017–2018	Clippers	Blake Griffin	PF	0.0171	0.0141	0.0201
8	2017–2018	Timberwolves	Karl-Anthony Towns	C	0.0160	0.0133	0.0188
9	2017–2018	Suns	Devin Booker	SG	0.0159	0.0129	0.0189
10	2017–2018	Thunder	Russell Westbrook	PG	0.0156	0.0118	0.0193
1	2018–2019	Pistons	Blake Griffin	PF	0.0235	0.0176	0.0294
2	2018–2019	Warriors	DeMarcus Cousins	C	0.0224	0.0194	0.0254
3	2018–2019	76ers	Joel Embiid	C	0.0213	0.0186	0.0241
4	2018–2019	Nuggets	Nikola Jokic	C	0.0213	0.0156	0.0270
5	2018–2019	Thunder	Paul George	SF	0.0212	0.0183	0.0241
6	2018–2019	Timberwolves	Karl-Anthony Towns	C	0.0208	0.0178	0.0237
7	2018–2019	Grizzlies	Marc Gasol	C	0.0199	0.0159	0.0240
8	2018–2019	Thunder	Russell Westbrook	PG	0.0184	0.0136	0.0232
9	2018–2019	Rockets	James Harden	SG	0.0183	0.0132	0.0234
10	2018–2019	Pelicans	Julius Randle	C	0.0181	0.0147	0.0215

High values of $\widetilde{\varvec{\gamma }}_{athlete}^{\tiny \text{(CS) }}$ indicate athletes who perform better than expected on their team within the season, values close to zero represent athletes who perform as expected, and high negative values represent athletes who perform worse than expected. However, it is important to note that a high consistency score does not necessarily mean that an athlete is the best player on their team or in that season. For example, DeMarcus Cousins had a much higher than expected consistency score for the Pelicans team in 2017–2018, but he is not necessarily the best athlete on the team or in the season, despite being listed as one of the 10 most relevant athletes in Table 3.

Russell, on the other hand, has consistently performed better than expected on his team since 2016–2017, where he ranked 6th with a Consistency Score of 0.0163 and a 95% confidence interval of [0.0134, 0.0193]. Therefore, the consistency score can also be used as a statistic to measure the performance of an investment strategy.

**Figure 2:**
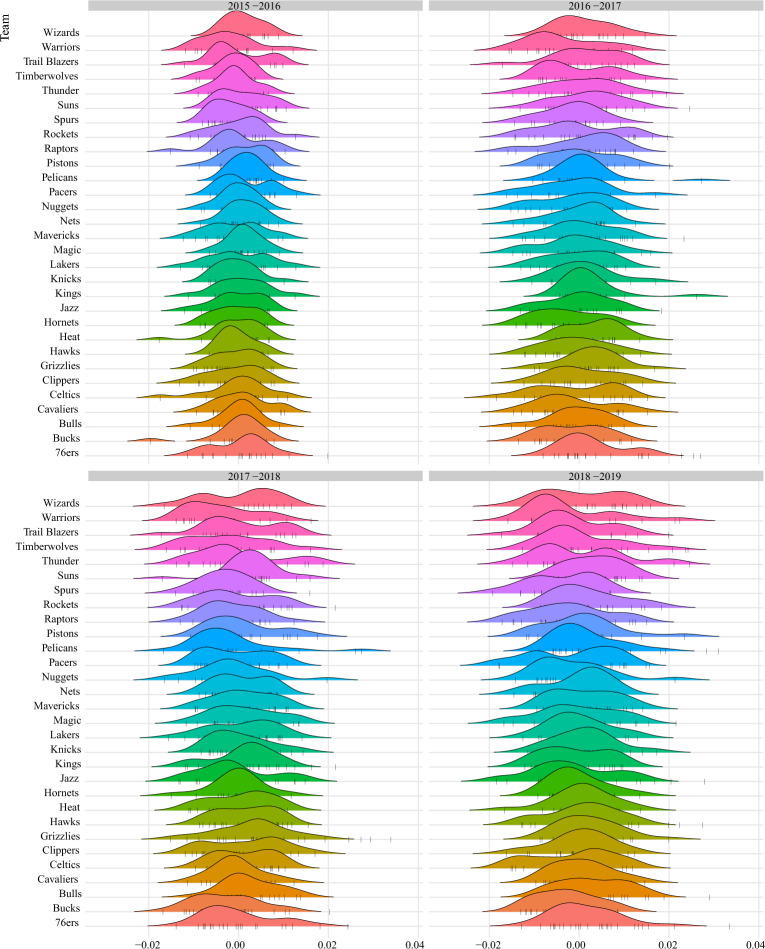
Density chart of $\widetilde{\varvec{\gamma }}_{athlete}^{\tiny \text{(CS) }}$ by a team within the season, with athletes represented by vertical bars. Values close to zero represent athletes whose performance is equal to the expected performance, while values below or above zero represent the worst or best performance, respectively, compared to the expected performance.

To better represent the $\widetilde{\varvec{\gamma }}_{athlete}^{\tiny \text{(CS) }}$ score, we can use the density plot in Fig. 2, which shows the distribution of score values by team for a given season.

The $\widetilde{\varvec{\gamma }}_{athlete}^{\tiny \text{(CS) }}$ score is a measure of an athlete’s performance compared to the expected performance within their team within the season. Athletes with a high positive score have performed better than expected, while athletes with a score close to zero have performed as expected and athletes with a high negative score have performed worse than expected. For example, DeMarcus Cousins performed much better than expected for the Pelicans team in the 2017–2018 season, but that does not necessarily make him the best athlete on the team or in the season, even if he is ranked in the top 10 most relevant athletes (Table 3). Another interesting example is James Harden, who ranked 34th in 2017–2018 with a value of 0.0120 [0.0092, 0.0148], but moved up 25 places in 2018–2019 with a value of 0.0181 [0.147, 0.0215] (Table 4). This suggests that Harden has tried harder and performed much better than expected in 2018–2019, which is directly reflected in his Relevance Score Index.

The consistency score can also be used as a measure of the performance of an investment strategy. A team with left-skewed density indicates that most athletes perform better than expected, while a right-skewed density indicates that most athletes perform less than expected. However, this measure cannot be used to compare teams because a team with a left-skewed density is not necessarily better than a team with a right-skewed density.

Take the Cavaliers team in the 2015–2016 season as an example, three of their athletes, Kevin Love, J.R. Smith and Channing Frye, performed much better than expected, while Tristan Thompson performed worse than expected while the other athletes on the team performed as expected.

A density plot of $\widetilde{\varvec{\gamma }}_{athlete}^{\tiny \text{(CS) }}$ scores by team and season is shown in Fig. 2. The standard deviation of the scores will be small if the athletes are close to their expected performance, resulting in scores close to zero. A right-skewed distribution indicates that most athletes perform less than expected, while a left-skewed distribution indicates that most athletes perform more than expected.

### Model performance

To evaluate the performance of our model without the need for a model update as new data are generated we compared the ON scores from a model fitted to a portion of the data to the ON scores from a model using the complete data. We first split the data into training and test sets as described in the Methods section. We then fitted the model to the training set and used this model to predict the ON scores in the test set. We then refitted the model using the complete data and compared the true (i.e. model based on the full data) and predicted ON values (i.e. model based on the test data) to calculate several performance measures: the root mean square error (RMSE), the concordance correlation coefficient (CCC), the Pearson correlation coefficient and the accuracy measure $\text{ C}_{b}$. The results are summarised in Table 5. We found low ON Score prediction bias for all scenarios, indicating that the model can calculate an athlete’s ON score with high accuracy ($\text{ C}_{b} > 0.99$) and precision ($r > 0.96$). In addition, we observed high agreement between the observed and predicted values ($\text{ CCC } > 0.95$) with narrow 95% confidence intervals. These results suggest that our model is a reliable tool for predicting ON scores for new games or seasons.

**Table 5:** Summary of predictive performance with the test set, the root mean square error (RMSE), the concordance correlation coefficient (CCC) and its 95% confidence interval, the Pearson correlation coefficient (*r*), the accuracy measure ($\text{ C}_{b}$), the number of observations in the test set ($N_{\text {Test}}$) and the number of observations in the training set ($N_{\text {Training}}$).

Test set	RMSE	CCC			r	$\text{ C}_{b}$	$N_{\text {Test}}$	$N_{\text {Training}}$
		${\hat{\rho }}_{CCC}$	Lower	Upper				
Season 2015–2016	$6.810 \times 10^{-4}$	0.9612	0.9603	0.9622	0.9615	0.9998	28,978	81,369
Season 2016–2017	$8.492 \times 10^{-4}$	0.9558	0.9548	0.9568	0.9631	0.9925	29,099	81,248
Season 2017–2018	$8.254 \times 10^{-4}$	0.9562	0.9553	0.9572	0.9627	0.9932	26,165	84,182
Season 2018–2019	$8.892 \times 10^{-4}$	0.9526	0.9515	0.9537	0.9598	0.9925	26,105	84,242
$10\times 4=40$ Games$^{*}$	$6.831 \times 10^{-4}$	0.9639	0.9569	0.9710	0.9647	0.9992	877	103,289
$50\times 4=200$ Games	$6.862 \times 10^{-4}$	0.9637	0.9597	0.9677	0.9643	0.9993	4,218	99,948
$150\times 4=600$ Games	$6.882 \times 10^{-4}$	0.9637	0.9617	0.9758	0.9644	0.9993	11,925	92,201

* $10 \times 4$ means a random sample of 10 games by each of 4 seasons, which represents 40 games

Thus, since we have shown that the predictions based on our proposed model are not biased, we can confidently use it to predict the ON score. This justifies the use of the model as an ’on the fly’ approach for calculating ON scores and removes the necessity to rerun the model after each game as opposed to an end of season update for example. Moreover, the prediction with the multilevel model is as fast as the calculation of the ON based on the PCA. The main difference, however, is that for our model we use five response variables measured during the game to make predictions, whereas with PCA the number of variables required increases to 22.

It is crucial to emphasize that the validation results also demonstrate the model’s efficiency in terms of time and applicability. The model’s ability to accurately predict ON scores without the need for frequent re-fitting with new data is a significant advantage. Although assuming a static covariance matrix has its limitations, our model’s consistent performance underscores its robustness and real-time applicability in sports analytics. Future efforts will focus on enhancing adaptability to changing game dynamics. The primary challenge is fitting complex models efficiently, especially when data is received faster than computational processing can accommodate.

**Figure 3:**
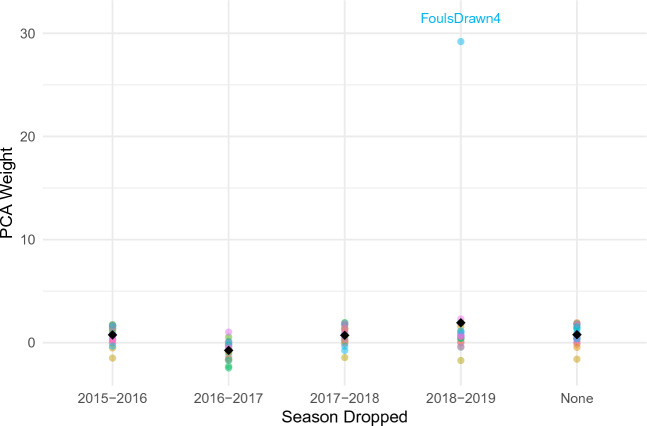
Variability of PCA weights with seasonal data exclusion in elite basketball performance analysis, where the black point represents the mean PCA weight.

The stability of PCA weights across different seasons can be seen in Fig. 3, as demonstrated by the minimal variation even with the exclusion of specific seasons, underscores the robustness and wide applicability of our model.

A notable exception is the variable ‘FoulsDrawn,’ which emerges as an outlier (Table 6). Although its behavior remains difficult to explain within the current data set based on previous years, we have opted to retain it in our analysis. This decision aligns with our commitment to a data-driven approach, ensuring that our findings are comprehensive and reflective of all observed variables.

**Table 6:** Mean and Standard deviation for ‘FoulsDrawn’ given the season.

Season	Mean	SD
2015–2016	0.0000	0.0000
2016–2017	0.0039	0.1363
2017–2018	0.0125	0.2416
2018–2019	1.9738	2.0597

The consistent pattern observed in PCA scores within each season, marked by negligible deviations, highlights the model’s ability to deliver consistent interpretations of performance. Moreover, our hierarchical analytical approach effectively captures the complex dynamics of team and player interactions, thereby enriching the interpretability of performance metrics. The model’s predictive prowess is further evidenced by low RMSE (Root Mean Square Error) and high CCC (Concordance Correlation Coefficient) values, signifying the PCA’s critical role in efficiently distilling predictive insights from diverse athlete performance metrics.

Finally, we have developed a user-friendly and interactive Shiny web application for our proposed model. The application can be accessed at https://doi.org/10.5281/zenodo.7787951. Our app provides a comprehensive set of features including descriptive analysis, model diagnostic, model prediction, and other important statistics. With this tool, users can easily explore and verify the results of our model in a convenient and efficient manner. We believe that our Shiny app will be a valuable resource for researchers and practitioners in the field.

## Comparison of ON with other metrics

In the scientific literature, the most commonly used metrics to evaluate individual athletes by team are plus-minus ratings, Athlete Efficiency Rating (PER) and the ESPN score called Real Plus-Minus (RPM). The adjusted plus-minus ratings were proposed by Rosenbaum^23^, who explicitly modelled the offensive and defensive ratings for each athlete using a linear regression approach^24^. In general, an athlete’s plus-minus is calculated as the difference between their team’s points and the opposing team’s points while they are in the game. RPM was developed by Ilardi^25^ using a Bayesian approach and extensive out-of-sample testing to improve the adjusted plus-minus. PER^26^, on the other hand, calculates the rating of an athlete’s performance per minute as the difference between the sum of their positive performances and the sum of their negative performances^26^.

However, none of these statistical methods take into account the hierarchical structure of the data. In contrast, the ON score proposed in this paper uses a multilevel regression model to account for the natural hierarchical structure of the data (athlete within team within season). In addition, the consistency score derived from the model provides valuable information on how much an athlete contributes to the team within a season, which cannot be determined using traditional metrics. This solves the issue of determining the contribution of individual athletes using plus-minus statistics, as discussed by Hvattum^3^.

Thus, coaches or managers could use the ON score and the consistency score as tools to assess the value of an athlete’s contribution to their team in a given season or over multiple seasons. In comparison, the plus-minus ratings, PER and RPM are useful but limited in their ability to address the hierarchical structure of the data and provide specific information about an athlete’s contribution within a team.

**Figure 4:**
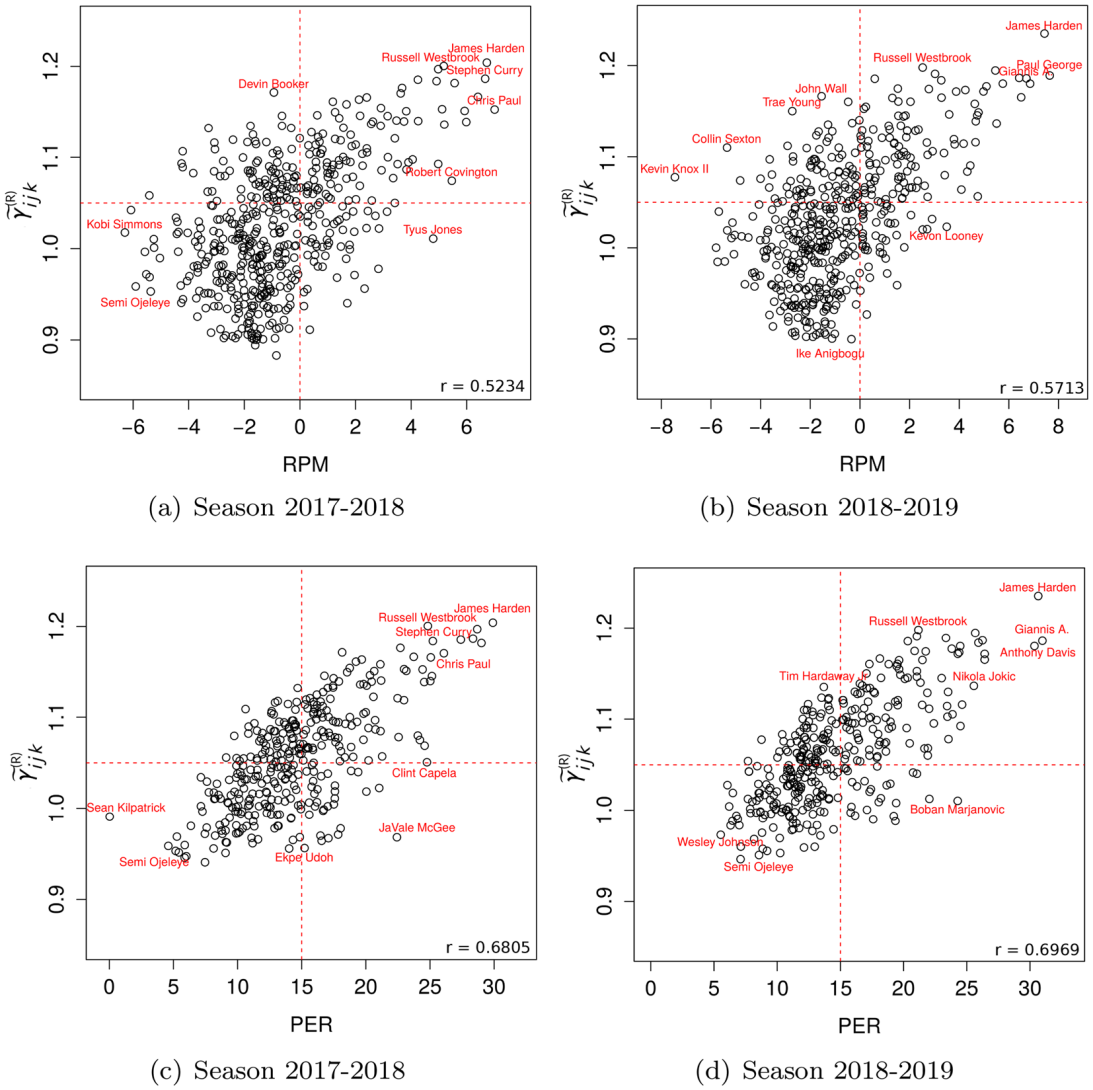
Comparison of Relevance Score ($\displaystyle \gamma ^{\tiny \text{(R) }}_{ijk}$) to RPM (**a**,**b**) and PER (**c**,**d**) by season.

Figure 4 shows the median relevance score, $\displaystyle \gamma ^{\tiny \text{(R) }}_{ijk}$, against RPM and PER for the seasons 2017–2018 and 2018–2019.

We note that our data set for the 2017–2018 and 2018–2019 seasons includes 534 and 514 athletes, respectively. However, we found that only 496 and 478 athletes had RPM and 342 and 346 athletes had PER for each of these seasons, respectively. We also found a moderate correlation between $\displaystyle \gamma ^{\tiny \text{(R) }}_{ijk}$ and RPM by season ($r=0.52$ and $r=0.57$) and a similarly moderate correlation between $\displaystyle \gamma ^{\tiny \text{(R) }}_{ijk}$ and PER by season ($r=0.68$ and $r=0.70$). However, the correlation between $\displaystyle \gamma ^{\tiny \text{(R) }}_{ijk}$ and PER was slightly higher than the correlation between $\displaystyle \gamma ^{\tiny \text{(R) }}_{ijk}$ and RPM, suggesting that the metric PER may not be completely context-agnostic, as discussed by Deshpande and Jensen^13^.

We should expect a moderate Pearson correlation between $\displaystyle \gamma ^{\tiny \text{(R) }}_{ijk}$ and PER, since the ON score also takes into account points, assists, rebounds, fouls and other performance criteria. This underlines the relevance of the ON score as an effective and context-sensitive measure for assessing athletes’ performance, especially when compared to more context-agnostic metrics such as RPM.

While we observed moderate values of Pearson correlation between relevance score and RPM or PER, we should also consider cases where these metrics do not match in terms of $\gamma ^{\tiny \text{(R) }}_{ijk}$. For example, in the 2017–2018 season, James Harden, Stephen Curry and Anthony Davis were found to have high values of $\displaystyle \gamma ^{\tiny \text{(R) }}_{ijk}$, PER and RPM, respectively, while in the 2018–2019 season Giannis Antetokounmpo, James Harden and Anthony Davis had higher values of these metrics.

On the other hand, we found discrepancies between the metrics when looking at Chris Paul’s performance. Based on RPM, Paul was ranked 1st, while based on PER and $\displaystyle \gamma ^{\tiny \text{(R) }}_{ijk}$, he was ranked 15th and 18th, respectively. This illustrates how RPM can make it challenging to compare athletes across teams, as discussed by Hvattum^3^. Using the $\varvec{\gamma }_{athlete}^{\tiny \text{(CS) }}$ score, we found that Chris Paul had a consistency score of $\widetilde{\varvec{\gamma }}_{athlete}^{\tiny \text{(CS) }}=0,0108$ ($[-0.0015, 0.0232]$), ranking third on the Rockets’ team behind James Harden and Trevor Ariza. Although Chris Paul’s contribution to the team was not significantly different from Harden’s and Ariza’s, he had higher variability in his contributions, as reflected in his lower rank based on $\displaystyle \gamma ^{\tiny \text{(R) }}_{ijk}$ and PER compared to RPM.

**Figure 5:**
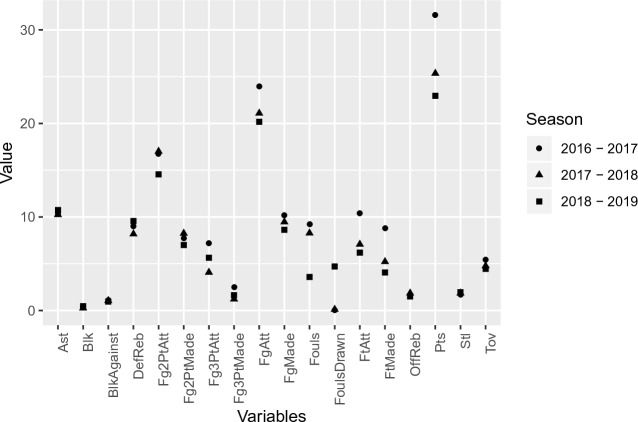
Russell Westbrook results for the seasons 2016–17, 2017–18, and 2018–19.

In contrast, Russell Westbrook’s performance in the 2018–2019 season was ranked 2nd, 35th and 42nd by $\displaystyle \gamma ^{\tiny \text{(R) }}_{ijk}$, PER and RPM respectively, so in this case RPM and PER were more in line with each other than with relevance value. Figure 5 illustrates Westbrook’s results in the 2016–17, 2017–18 and 2018–19 seasons. In the 2018–19 season, he had fewer offensive scores for Fg, Fg2Pt and Ft compared to the other seasons, but had his highest number of assists (10.7), fouls drawn and steals, combined with his lowest number of fouls and turnovers that season.

The relevance score in this case is an indication that Westbrook had a more technically impressive season in 2018-2019, as he drew more fouls. This metric reflects an effective way for a team to gain an advantage and score more points or get opposing athletes into foul trouble, potentially forcing them out of the game. Furthermore, the estimates of the consistency values and corresponding $95\%$ confidence intervals for Westbrook in these seasons were 0.0163 ($\text{ CI}_{95\%} [0.0134, 0.0193]$), 0.0156 ($\text{ CI}_{95\%} [0.0118, 0.0193]$) and 0.0184 ($\text{ CI}_{95\%} [0.0136, 0.0232]$), suggesting that Westbrook is consistently performing above expected levels for his team, with the 2018-19 estimate being higher. Taken together, then, $\displaystyle \gamma ^{\tiny \text{(R) }}_{ijk}$ and $\varvec{\gamma }_{athlete}^{\tiny \text{(CS) }}$ provide a more comprehensive assessment of an athlete’s performance. It is important to note that coaches may also consider the use of RPM and PER to inform their decisions.

### ON and impact score

The Impact Score, introduced in 2016 by Deshpande and Jensen^13^, is a popular method of evaluating athletes that uses linear Bayesian regression to create a single ranking of all athletes in the league based on a full posterior analysis. While the authors show that the posterior distribution of teams can be useful to take into account the contributions of athletes within their team, our Consistency Score presented in the Methods section offers a different approach to evaluating athletes’ performance that also takes into account their contribution to the team.

A notable advantage of our approach is the use of classical restricted likelihood methods proposed by Laird and Ware in 1982, which allow for faster computation than the Bayesian framework used by Deshpande and Jensen^13^. Our model predictions are also suitable for use during a match, allowing coaches and clubs to calculate the time specific adjusted ON score in just a few seconds.

Another important difference between the two approaches is the inference for each athlete. Deshpande and Jensen^13^ showed that their 1000 posterior samples used to calculate the 95% credible intervals for each athlete’s partial effects-based rank had a wide range, with the 95% credible interval for LeBron James’ rank being [3, 317]. In contrast, the 10,000 Monte Carlo simulations from the Consistency Score posterior distribution using empirical Bayes inference showed narrower 95% confidence intervals than those found by Deshpande and Jensen (2016)^13^, as shown in Table 4. Overall, both the impact score and the consistency score can provide useful information for assessing athlete performance, but they offer different approaches to achieving this goal.

## Final remarks and future work

This paper has introduced a transformative methodology for sports analytics, applicable across various sports, with a particular focus on basketball. By integrating hierarchical data structures and developing a novel consistency score, we have demonstrated a method that significantly enhances understanding of athlete contributions within teams. The low bias of the ON model and the high precision and accuracy of our predictions make our methodology a powerful tool that can substantially reduce the number of predictors to be recorded during a game.

Furthermore, the approach presented in this paper is applicable to all sports where a valid composite metric is required which is adjusted for the hierarchical structure of the data (i.e. player within position with team over time) using a multilevel regression model. The composite could be based on performance metrics, as in the NBA example presented, or indeed for workload metrics if a composite is required for assessing a players overall workload based on training load or in game workload metrics (e.g. distance covered, high intensity running etc).

In conclusion, our study marks a significant advancement in sports analytic, particularly for basketball. Future research should integrate expert assessments from coaches and players for qualitative validation of the ON score methodology. Longitudinal analyses correlating ON scores with career metrics, along with cross-sport model adaptation, will further test the model’s applicability and highlight the importance of including hierarchical structures. Additionally, developing sport-specific hierarchical models and adapting plus-minus statistics for diverse data structures will refine our approach.

## Methods

### Data characteristics

The dataset utilised in this analysis was procured from the Basketball Reference database (https://www.basketball-reference.com/), a publicly accessible source of NBA data. The comprehensive database that resulted from this compilation is available at https://doi.org/10.5281/zenodo.8056757/. This database encompasses the Win/Loss records for all thirty teams, box scores and more advanced statistics. During a typical season, each team participates in 82 regular-season matches, culminating in a sum of 1230 games played. Consequently, with four seasons incorporated in the dataset, we account for 4920 regular-season matches available for examination.

A notable characteristic of this dataset is the varying quantity of repeated measurements per athlete within a team across a season. This enables the exploration of player performance within a team and for those that have moved to a different team. Nevertheless, it is crucial to recognise that these repeated measurements are not independent, and statistical methods such as hierarchical modelling must be employed to handle the data appropriately.

Furthermore, it is crucial to take into account potential biases or limitations within the dataset, in addition to its size and format. For instance, the Basketball Reference website might not incorporate data from specific matches or may exclude particular variables, potentially impacting the quality and accuracy of the dataset. Moreover, the dataset might not encompass information on certain types of players or teams, which could constrain the generalisability of the findings. Acknowledging these limitations is essential for ensuring the validity and reliability of the analyses conducted using this data.

### ON score

**Figure 6:**
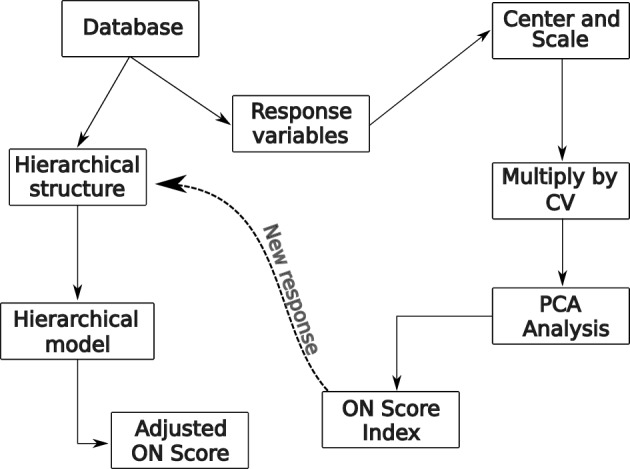
Flow chart for the construction of the adjusted ON index.

To develop the ON Score, we employ a two-stage methodology. In the initial stage, we utilise principal component analysis (PCA) to compute the preliminary ON index. Subsequently, in the second stage, we adjust this initial index with a hierarchical model, culminating in the final ON Score. The entire process is depicted in Fig. 6.

During the first stage, we commence with a vector $\displaystyle {\textbf{r}} = \left[ r_{1}, r_{2}, \ldots , r_{n}\right] ^{\top }$ comprising continuous response variables, some of which might be correlated. We compute their sample means ${\bar{\textbf{r}}}=\left[ {\bar{r}}_{1}, {\bar{r}}_{2}, \ldots , {\bar{r}}_{n}\right]$ and sample standard deviations ${\bar{\textbf{s}}}=\left[ {\bar{s}}_{1}, {\bar{s}}_{2}, \ldots , {\bar{s}}_{n}\right]$ to standardise each element of ${\textbf{r}}$ using the relevant sample standard deviation and mean. This results in a new set of variables ${\textbf{z}}=\left[ z_1,z_2,\ldots ,z_{n}\right]$ possessing zero mean and unit variance.

Nevertheless, PCA is not scale-invariant, and utilising the covariance matrix of ${\textbf{z}}$, equivalent to the correlation matrix of ${\textbf{r}}$, assigns equal weight to all response variables. This could produce unrealistic results, for instance, if the highest-ranked athlete does not score in a match and the lowest-ranked athlete achieves the highest score. In such cases, applying PCA based on the correlation matrix of ${\textbf{r}}$ results in equivalent scores for both athletes, which is unrealistic.

To address this issue, we can multiply each element of ${\textbf{z}}$ by its respective coefficient of variation (CV), a measure of the dispersion of a probability distribution, defined as the ratio of the standard deviation to the mean. Scaling by CV ensures that the variability of each response variable is suitably accounted for. The adjusted score ${\textbf{a}}=\left[ a_1,a_2,\ldots ,a_n\right]$ is obtained by multiplying each element of ${\textbf{z}}$ by the corresponding CV. However, caution must be exercised when applying this approach to response variables where ${\bar{r}}_{i}=0$ or when ${\bar{r}}_{i}$ approaches zero. In such instances, $z_{i}^{*}$ becomes infinite, potentially causing issues in the analysis. Therefore, we need to address such cases separately. Specifically, we can define

$$\begin{aligned} z_{i}^{*} = z_{i} \frac{s_{i}}{{\bar{r}}_{i}} = \frac{r_{i} - {\bar{r}}_{i}}{s_{i}} \frac{s_{i}}{{\bar{r}}_{i}} = \frac{r_{i} - {\bar{r}}_{i}}{{\bar{r}}_{i}}, \quad |{\bar{r}}_{i}|>0. \end{aligned}$$

This ensures that the adjusted score remains well-defined, avoiding any issues that might arise from infinite values of $z_{i}^{*}$.

Let ${\textbf{y}}^{\top }=\left[ {y_{1}, y_{2}, \ldots , y_{n}}\right]$ represent a set of uncorrelated variables, each being a linear combination of the ${\textbf{z}}^{\top }$ variables, expressed as

$$\begin{aligned} \displaystyle y_{c} = \alpha _{c1} z^{*}_{1} + \alpha _{c1} z^{*}_{2} + \ldots + \alpha _{cn} z^{*}_{n}, \quad c = 1, 2, \ldots , n. \end{aligned}$$

The *c*-th principal component (PC) is the linear combination $\displaystyle y_{c}=\varvec{\alpha }_{c}^{\top }{\textbf{z}}^{*}$ that possesses the largest sample variance, subject to the conditions $\varvec{\alpha }_{c}^{\top }\varvec{\alpha }_{c}=1$ and $\varvec{\alpha }_{c}^{\top }\varvec{\alpha }_{c'}=0, \quad c'<c$. Let ${\textbf{A}}$ denote an $n \times n$ matrix containing initial vectors that define the *n* principal components, ${\textbf{A}}=[\varvec{\alpha }_{1}, \varvec{\alpha }_{2}, \ldots , \varvec{\alpha }_{n}]$, and $\varvec{\lambda }^{\top }=[\lambda _{1}, \lambda _{2}, \ldots , \lambda _{n}]$ a vector of length *n* containing their corresponding variances (eigenvalues).

In our methodology, we utilize all the components derived from the Principal Component Analysis (PCA). This comprehensive approach allows us to capture the entire spectrum of variability within the data set. Each PCA component contributes to the construction of the ON score, ensuring that no significant aspect of the athletes’ performance data is overlooked. By incorporating all PCA components, the ON score becomes a multi-dimensional vector that holistically represents athlete performance, providing a nuanced and thorough evaluation.

Utilising ${\textbf{A}}$ and $\varvec{\lambda }$, we can calculate a weighted matrix ${\textbf{W}}$ as

1 $$\begin{aligned} {\textbf{W}} = \left( {\textbf{1}}_{n}^{\top } \otimes \varvec{\lambda }\right) \odot {\textbf{A}}^{\top }S^{-1}, \text{ with } S = \sum _{c=1}^{n} \lambda _{c}, \end{aligned}$$

where ${\textbf{1}}$ is a vector of ones; $\otimes$ is the generalised Kronecker product of two arrays; $\odot$ represents the Hadamard product, which is both associative and distributive over addition. Equation (1) demonstrates the weighted mean of a set $\varvec{\alpha }_{i}={\alpha _{c1}, \alpha _{c2}, \ldots , \alpha _{cn}}$ with corresponding non-negative weights $\varvec{\lambda }^{\top }=\left[ {\lambda _{1}, \lambda _{2}, \ldots , \lambda _{n}}\right]$. Consequently, the principal components with high variances contribute more to the weighted mean than those accounting for low variances.

Finally, the ON $\left( \varvec{\gamma }^{(S)}\right)$ vector, which encompasses all scores for games and athletes, is depicted as a *K*-dimensional vector and is expressed as follows:

$$\begin{aligned} \varvec{\gamma }^{(S)} = \left( {\textbf{W}}^{\top }{\textbf{1}}_{n}\right) {\textbf{Z}}^{*\top }, \end{aligned}$$

where ${\textbf{Z}}^{*}=[{\textbf{z}}_{1}^{*}, {\textbf{z}}_{2}^{*}, \ldots , {\textbf{z}}_{n}^{*}]$ is a $K \times n$ matrix of responses used in the PCA analysis.

**Figure 7:**
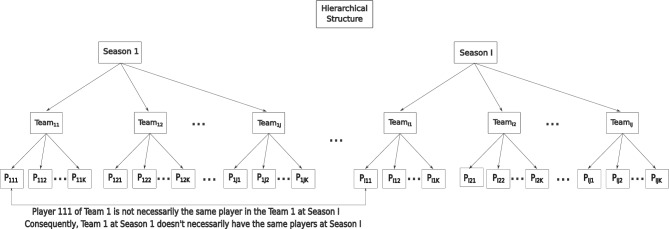
Hierarchical structure of basketball database.

In the second stage, $\gamma ^{(S)}{ijkl}$ denotes the observed score for the *i*-th season ($i=1,2,\ldots ,I$), the *j*-th team ($j=1,2,\ldots , J$), the *k*-th athlete ($k=1,2,\ldots , K$), and the *l*-th repeated measure of athlete *k* within team *j* and season *i* ($l=1,2,\ldots ,L_{ijk}$) (Fig. 7). The existence of numerous repeated measurements for athletes in both teams and seasons results in correlated errors that must be considered in the model assumptions, alongside the hierarchical structure of the data.

Multilevel models are particularly advantageous in this scenario, as they adeptly model complex hierarchical structures, manage missing data effectively, and provide more interpretable fixed effects and variance components parameters in comparison to conventional repeated measures models^19^. We posit that the regressor variables $f_{ijkl1}, f_{ijkl2}, \ldots , f_{ijklM}$ are fixed explanatory variables, measured without error, representing the fixed effects of venue (Home or Away), athlete’s position, rookie status, among others. Additionally, we assume that $t_{ijkl1}, t_{ijkl2}, \ldots , t_{ijklP}$ are continuous covariates assessed during the match. Thus, we may write the three-level multilevel model as follows:

2 $$\begin{aligned} \begin{array}{c} \displaystyle \gamma ^{\tiny \text{(S) }}_{ijkl} = \beta _0 + \sum _{m=1}^{M}\beta _{m} f_{ijklm} + \sum _{p=1}^{P}\sum _{q=1}^{Q_{p}}\theta _{pq}\text{ P}_{pq}\left( t_{ijklp}\right) + b_{i} + b_{i,j} + b_{ij,k} + \epsilon _{ijkl} \\ \\ \displaystyle b_{i} \sim \text{ N }\left( 0,\sigma _{1}^{2}\right) , \quad b_{i,j} \sim \text{ N }\left( 0,\sigma _{2}^{2}\right) , \quad b_{ij,k} \sim \text{ N }\left( 0,\sigma _{3}^{2}\right) , \quad \epsilon _{ijk} \sim \text{ N }\left( 0,\sigma ^{2}\right) \end{array} \end{aligned}$$

where $\beta _0$ is the intercept, ${\textbf{f}}_{ijkl}=\left[ f_{ijkl1}, f_{ijkl2}, \ldots , f_{ijklM}\right]$ are factor variables with $m=1,2,\ldots ,M$, $\text{ P}_{pq}(\cdot )$ is the *q*-th order orthogonal polynomial for the *p*-th continuous variable $t_{ijklp}$ with $p=1,2,\ldots , P$ and $q=1,2,\ldots , Q_{p}$, $\varvec{\beta }=\left[ \beta _1, \beta _2, \ldots , \beta _{M}\right] ^{\top }$, and $\varvec{\theta }_{p}=\left[ \theta {p1}, \theta _{p2}, \ldots , \theta _{pQ_{p}}\right]$ are unknown fixed-effect parameters. Additionally, $b_i$ represents season random effects, $b_{i,j}$ represents team within season random effects, $b_{ij,k}$ represents athlete within team within season random effects, and $\epsilon _{ijk}$ denotes the within-group errors, which are assumed to be independent and identically distributed with mean zero and variance $\sigma ^2$. Furthermore, all random effects are assumed to be independent of each other and independent of the within-groups error. The model specification (2) can be expressed in matrix notation as:

3 $$\begin{aligned} \begin{array}{c} \varvec{\gamma }^{\tiny \text{(S) }}_{ijk} = {\textbf{X}}_{ijk}\varvec{\beta }^{*} + {\textbf{z}}_{i,jk}b_{i}+{\textbf{z}}_{ij,k}b_{ij}+{\textbf{z}}_{ijk}b_{ijk} +\varvec{\epsilon }_{ijk}, \\ \\ \displaystyle b_{i} \sim \text{ N }\left( 0,\sigma _{1}^{2}\right) , \quad b_{i,j} \sim \text{ N }\left( 0,\sigma _{2}^{2}\right) , \quad b_{ij,k} \sim \text{ N }\left( 0,\sigma _{3}^{2}\right) , \quad \varvec{\epsilon }_{ijk} \sim \text{ N }\left( 0,\sigma ^{2}{\textbf{I}}\right) \end{array} \end{aligned}$$

where ${\textbf{z}}_{i,jk}$ is a $L_{ijk}$-dimensional vector for the random effects of season on measurements for team *j* and athlete *k* within team; ${\textbf{z}}_{ij,k}$ is a $L_{ijk}$-dimensional vector for the random effects of team *j* within season *i* on measurements for athlete *k*; and ${\textbf{z}}_{ijk}$ is a $L_{ijk}$-dimensional vector for the random effects for athlete *k* within team *j* and season *i*. As a consequence of the hierarchical structure, we can write ${\textbf{z}}_{i,jk} = {\textbf{z}}_{ij,k} = {\textbf{z}}_{ijk} = \left[ 1, 1, \ldots , 1\right] ^T$.

Furthermore, $b_{i}$, $b_{ij}$, and $b_{ijk}$ are random effects of season, team within season, and athlete within team and season, respectively; and $\varvec{\epsilon }_{ijk}$ is the vector of within-group errors. Here, ${\textbf{X}}_{ijk}$ should be viewed as a partitioned matrix given by

$$\begin{aligned} {\textbf{X}}_{ijk} = \left[ \begin{array}{c|c|c} {\textbf{1}}&{\textbf{F}}_{ijk}&{\textbf{P}}_{ijk} \end{array}\right] \end{aligned}$$

where ${\textbf{1}}$ represents a vector of ones related to the overall mean ($\beta _0$); ${\textbf{F}}_{ijk}$ is the design matrix containing fixed effects of factor variables, which are categorical ones that can be either numeric or string variables; ${\textbf{P}}_{ijk}$ is an orthogonal matrix built using the Gram-Schmidt process to form orthogonal polynomials for each of the *p*-th continuous covariate. Clearly, the matrix ${\textbf{X}}_{ijk}$ can be extended to accommodate interaction effects between explanatory variables. Furthermore, $\varvec{\beta }^{}$ should be viewed as a vector containing all fixed effects parameters, that is, $\varvec{\beta }=\left[ \varvec{\beta }^{\top }, \varvec{\theta }^{\top }\right] ^{\top }$. Therefore, in terms of the hierarchical model, the conditional multilevel model can be written as $\varvec{\gamma }^{\tiny \text{(S) }}_{ijk}|b_{i,jk},b_{ij,k},b_{ijk} \sim \text{ N }\left( {\textbf{X}}_{ijk}\varvec{\beta }^{*}+{\textbf{z}}_{i,jk}b_{i}+{\textbf{z}}_{ij,k}b_{ij}+{\textbf{z}}_{ijk}b_{ijk}, \sigma ^2{\textbf{I}}\right)$, and then marginally $\varvec{\gamma }^{\tiny \text{(S) }}_{ijk} \sim \text{ N }\left( {\textbf{X}}_{ijk}\varvec{\beta }^{*}, {\textbf{V}}_{ijk}\right)$, where ${\textbf{V}}_{ijk}=\sigma ^2_{1}{\textbf{z}}_{i,jk}{\textbf{z}}_{i,jk}^{\top }+\sigma ^2_{2}{\textbf{z}}_{ij,k}{\textbf{z}}_{ij,k}^{\top }+\sigma ^2_{3}{\textbf{z}}_{ijk}{\textbf{z}}_{ijk}^{\top } + \sigma ^2{\textbf{I}}_{L{ijk}}$.

### Estimation and model selection

To estimate the parameters in the multilevel model described by equation (3), we utilized a mixed-effect model equation. This equation allowed us to estimate both fixed and random effects for a given $\varvec{\beta }^{*}$ and $\varvec{\kappa }$. The vector $\varvec{\kappa }$ contains all the variance components of the model. The method we employed for estimation, which involves best linear unbiased prediction (BLUP) and best linear unbiased estimation (BLUE)^27,28^. To take into account the loss of degrees of freedom resulting from the adjustment of fixed effects, we used the constrained maximum likelihood (REML) method^28^. This approach modifies the maximum likelihood method (ML) by constraining the likelihood function to the linear unbiased fixed effects estimate. By doing so, we were able to estimate the variance components while removing the bias that occurs when the fixed effects and variance components are estimated simultaneously using the ML estimate. The REML method allowed us to obtain unbiased estimates of the variance components, which were used to estimate the random effects through the BLUP method. Finally, the fixed effects were estimated using the BLUE method.

For the model selection procedure, we start with a saturated parameter specification for fixed effects that takes into account main and interaction effects. Refinement of the fixed effects should only take place after a satisfactory variance-covariance structure has been selected. Taking into account the principle of parsimony, we aim for a variance-covariance structure with a small number of unknown parameters, but not too simple, as this may increase type I error rates, and not too complex, as this may reduce the test power (increasing type II error) in the selection of fixed effects^29^. Moreover, a highly complex structure requires the estimation of many unknown parameters, which could significantly complicate the interpretation of the variance-covariance pattern and provide poor predictions for the adjusted ON score.

We employ a top-down strategy for model selection^30^, using likelihood ratio tests for nested models. We examine a series of nested models with varying variance-covariance structures, commencing with a simpler model and progressively increasing complexity. At each stage, we compare the simpler model’s likelihood with that of the more complex model through a likelihood ratio test. If the more complex model offers a significant improvement in fit compared to the simpler model, we accept it as the best-fitting model. This process continues until we identify a model that does not provide a significant improvement in fit over its predecessor. It is essential to note that the likelihood ratio test is particularly well-suited for this process, as it is specifically designed for comparing nested models.

Lastly, we fine-tune the fixed effects of the chosen model using stepwise regression or Bayesian model selection techniques, such as the Bayesian Information Criterion (BIC) or the Deviance Information Criterion (DIC). These methods enable us to select the most pertinent fixed effects while preventing overfitting.

### The intraclass correlation coefficient

The intraclass correlation coefficient (ICC) is a valuable measure to account for the degree of similarity among units within each group, and it can be readily calculated within the context of mixed-effects models. The ICC ranges between 0 and 1, where values close to zero signify high similarity between units, and values near 1 indicate low similarity among units in the same group. Various intraclass correlation coefficients can be derived from the model (3), but in this instance, we are seeking the ICC expression to evaluate the cumulative proportion of variability attributable to the season, the team within the season, and the athlete within the team within the season concerning the total variability of the data. Under this scenario, the ICC under model (3) is given by

4 $$\begin{aligned} ICC_{1}&= \displaystyle \frac{\sigma _{1}^2}{\sigma _{1}^2+\sigma _{2}^2+\sigma _{3}^2+\sigma ^2}; \quad ICC_{2} = \displaystyle \frac{\sigma _{1}^2+\sigma _{2}^2}{\sigma _{1}^2+\sigma _{2}^2+\sigma _{3}^2+\sigma ^2}; \quad ICC_{3} = \displaystyle \displaystyle \frac{\sigma _{1}^2+\sigma _{2}^2+\sigma _{3}^2}{\sigma _{1}^2+\sigma _{2}^2+\sigma _{3}^2+\sigma ^2}, \end{aligned}$$

where $\text{ ICC}_1$, $\text{ ICC}_2$, and $\text{ ICC}_3$ represent the ICC for the season, the team within the season, and the athlete within the team within the season, respectively. Since the ICCs in expression (4) are functions of variance components, they can be estimated by substituting $\sigma _{1}^2$, $\sigma _{2}^2$, $\sigma _{3}^2$, and $\sigma ^2$ with their REML estimates. The ICC offers crucial information about the proportion of variance attributable to the different levels of the model, which is essential for understanding the proportion of variation in the outcome ascribed to each level. A high ICC value indicates that the majority of the variation in the outcome is explained by that level, while a low ICC value suggests that other sources of variation may be more significant.

### The consistency score index based on random effects

The Consistency Score (CS) offers valuable insights into the performance levels of athletes within their respective teams and seasons, signifying whether they surpass, meet, or fall short of their expected levels. By incorporating random effects, we can predict the CS and pinpoint which seasons, teams within a season, or athletes within a team exhibit performance levels that deviate from anticipated levels. In this manner, we can determine which athletes in a particular team excel or underperform compared to their teammates in a given season, or which teams exceed or fall short of their expected values throughout the season.

Moreover, the CS in model (3) can be employed to scrutinise team- or athlete-specific progress across seasons as an indicator of consistency in performance. We can compute the CS by season as follows:

$$\begin{aligned} \widetilde{\varvec{\gamma }}_{Season}^{\tiny \text{(CS) }}&=\text{ E }\left[ b_{i}|\varvec{\gamma }^{\tiny \text{(S) }}_{i}\right] = \text{ Cov }\left( b_i,\varvec{\gamma }^{\tiny \text{(S) }}_{i}\right) \text{ Var }\left( \varvec{\gamma }^{\tiny \text{(S) }}_{i}\right) ^{-1}\left( \varvec{\gamma }^{\tiny \text{(S) }}_{i}-\text{ E }\left( \varvec{\gamma }^{\tiny \text{(S) }}_{i}\right) \right) \\&= {\widehat{\sigma }}_{1}^2{\textbf{z}}_{i}^{\top }\hat{{\textbf{V}}}_{i}^{-1}\left( \varvec{\gamma }^{\tiny \text{(S) }}_{i} - {\textbf{X}}_{i}\hat{\varvec{\beta }^{*}}\right) , \end{aligned}$$

and by team within season as

$$\begin{aligned} \widetilde{\varvec{\gamma }}_{Team}^{\tiny \text{(CS) }} =\text{ E }\left[ b_{ij}|\varvec{\gamma }^{\tiny \text{(S) }}_{ij}\right] = {\widehat{\sigma }}_{2}^2{\textbf{z}}_{ij}^{\top }\hat{{\textbf{V}}}_{ij}^{-1}\left( \varvec{\gamma }^{\tiny \text{(S) }}_{ij} - {\textbf{X}}_{ij}\hat{\varvec{\beta }^{*}}\right) , \end{aligned}$$

and by athlete within team in a season as

$$\begin{aligned} \widetilde{\varvec{\gamma }}_{athlete}^{\tiny \text{(CS) }} =\text{ E }\left[ b_{ijk}|\varvec{\gamma }^{\tiny \text{(S) }}_{ijk}\right] = {\widehat{\sigma }}_{3}^2{\textbf{z}}_{ijk}^{\top }\hat{{\textbf{V}}}_{ijk}^{-1}\left( \varvec{\gamma }^{\tiny \text{(S) }}_{ijk} - {\textbf{X}}_{ijk}\hat{\varvec{\beta }^{*}}\right) . \end{aligned}$$

The vectors and matrices involved in the calculation of the Consistency Score (CS) index are important to understand for practical applications. Specifically, $\widetilde{\varvec{\gamma }}_{Season}^{\tiny \text{(CS) }}$ is an *n*-dimensional vector, and the matrices ${\textbf{V}}_{i}$, ${\textbf{V}}_{ij}$ and ${\textbf{V}}_{ijk}$ can be visualised in “Supplementary Material”. By analysing these matrices, we gain insight into the importance of random effects in the model and the variability of the data.

Furthermore, the adjusted ON score can be computed for each athlete based on their position in the game, utilising the $\widetilde{\varvec{\gamma }}_{athlete}^{\tiny \text{(CS) }}$ in conjunction with the fixed effect of position or other combinations of fixed and/or random effects that may be pertinent to explain specific intriguing patterns. This offers a robust tool for analysing performance data and pinpointing areas for enhancement.

Building upon this analysis, by employing simulations under model (3) we can capture the inferential uncertainty for $\widetilde{\varvec{\gamma }}_{Season}^{\tiny \text{(CS) }}$, $\widetilde{\varvec{\gamma }}_{Team}^{\tiny \text{(CS) }}$, and $\widetilde{\varvec{\gamma }}_{athlete}^{\tiny \text{(CS) }}$ as well as we can translate them into predictions for new games. The $100\left( 1-\alpha \right) \%$ confidence intervals for $\varvec{\gamma }_{Season}^{\tiny \text{(CS) }}$, $\varvec{\gamma }_{Team}^{\tiny \text{(CS) }}$ and $\varvec{\gamma }_{athlete}^{\tiny \text{(CS) }}$ are thus based on 10.000 Monte Carlo simulations from their posterior distributions using empirical Bayes (EB) inference for random effects^18^. This comprehensive approach allows for a deeper understanding of the performance dynamics and facilitates more accurate predictions for future games.

### Ratings athletes based on conditional expectation

We now describe how to rank athletes based on the conditional expectation under model (3). Specifically, we denote $E(\varvec{\gamma }^{\tiny \text{(S) }}_{ijk}|b_{i,jk},b_{ij,k}, b_{ijk}) = \varvec{{\widehat{\gamma }}}^{\tiny \text{(S) }}_{ijk}$ as an $L_{ijk}$-dimensional vector containing all conditional expectation values for the *k*-th athlete within team *j* in season *i*, ordered as $\zeta _{ijk1} = \min _{l} {\widehat{\gamma }}^{\tiny \text{(S) }}_{ijkl}< \zeta _{ijk2}< \ldots< \zeta _{ijk(L_{ijk}-1)} < \zeta _{ijkL_{ijk}} = \max _{l} {\widehat{\gamma }}^{\tiny \text{(S) }}_{ijkl}$. Since the average value can be strongly influenced by poor or exceptional performance by an athlete in a certain game, we can use the median value as an alternative measure because it is more robust to outliers. Thus, the median relevance score, ${\widetilde{\gamma }}^{\tiny \text{(R) }}_{ijk}$, is defined by

5 $$ {\widetilde{{\gamma}}} _{{ijk}}^{{\tiny{\text{(R) }}}}  = \left\{ {\begin{array}{*{20}c}    {\zeta _{{ijk\left( {\frac{{L_{{ijk}}  + 1}}{2}} \right)}} ,} & {{\text{if }}L_{{ijk}} {\text{ is  odd}}}  \\    {\frac{1}{2}\left[ {\zeta _{{ijk\left( {\frac{{L_{{ijk}} }}{2}} \right)}}  + \zeta _{{ijk\left( {1 + \frac{{L_{{ijk}} }}{2}} \right)}} } \right],} & {{\text{if }}L_{{ijk}} {\text{ is  even}}}  \\   \end{array} } \right. $$

The Wilcoxon signed rank test^31^ can be used to compute a symmetric two-sided $100\left( 1-\alpha \right) \%$ confidence interval for ${\widetilde{\gamma }}^{\tiny \text{(R) }}_{ijk}$. This interval can be expressed as $\left( {\widetilde{\gamma }}^{\tiny \text{ Lower }}_{ijk}, {\widetilde{\gamma }}^{\tiny \text{ Upper }}_{ijk}\right)$, where $P_{{\widetilde{\gamma }}^{\tiny \text{(R) }}_{ijk}}\left( {\widetilde{\gamma }}^{\tiny \text{ Lower }}_{ijk}< {\widetilde{\gamma }}^{\tiny \text{(R) }}_{ijk} < {\widetilde{\gamma }}^{\tiny \text{ Upper }}_{ijk}\right) =1-\alpha$ for all ${\widetilde{\gamma }}^{\tiny \text{(R) }}_{ijk}$. To obtain this interval, we first compute the value of $C_{ijk}$ as follows:

$$\begin{aligned} C_{ijk} = \frac{L_{ijk}\left( L_{ijk}+1\right) 
}{2}+1-t_{\alpha /2}. \end{aligned}$$

Here $t_{\alpha /2}$ is the upper $(\alpha /2)$-th percentile point of the null distribution of the test statistic $T^{+}_{ijk}=\displaystyle \sum _{l=1}^{L_{ijk}}R_{ijkl}\psi _{ijkl}$. In this expression, $\psi _{ijkl}$ takes the value 1 if $\zeta _{ijkl} > 0$, and 0 otherwise, and $R_{ijkl}$ denotes the rank of $|\zeta _{ijkl}|$, where $l=1,2,\ldots ,L_{ijk}$ in order of absolute value. The product $R_{ijkl}\psi _{ijkl}$ represents the positive signed rank of $\zeta _{ijkl}$ which takes the value 0 when $\zeta _{ijkl}$ is negative and the rank of $|\zeta _{ijkl}|$ when $\zeta _{ijkl}$ is positive.

Further, we set ${\widetilde{\gamma }}^{\tiny \text{ Lower }}_{ijk} = \zeta ^{\left( C_{ijk}\right) }_{ijk}$ and ${\widetilde{\gamma }}^{\tiny \text{ Upper }}_{ijk} = \zeta ^{\left( M+1-C_{ijk}\right) }_{ijk}= \zeta ^{\left( t{\alpha /2}\right) }_{ijk}$, where $M=\frac{L_{ijk}\left( L_{ijk}+1\right) }{2}$. This gives a confidence interval for ${\widetilde{\gamma }}^{\tiny \text{(R) }}_{ijk}$ that is symmetric and two-sided. The same approach can be used to rank other variables, such as teams, athletes by position and rookies.

### Model validation and predictions

When modelling athletes’ performance, it is essential to consider the complex relationships between athletes during a game. It is a common practice to assume that athletes within a team are not independent and exhibit constant variance on repeated measures. However, it is also reasonable to expect that the covariance between athletes may not be zero. In model 3, we assume a simplified structure for the variance-covariance matrix, including a constant correlation between athletes within the same team and a constant correlation between athletes from different teams. Nevertheless, it is crucial to explore more complex structures while avoiding overfitting and maintaining a parsimonious model.

Our second modelling assumption is that the conditional errors follow a Gaussian distribution with constant variance. This assumption is widespread in linear mixed models and can aid in interpretation and prediction^19^. However, the normality assumption may not always be suitable for certain datasets. Thus, it is crucial to evaluate the residuals’ distribution and consider alternative distributions if necessary^19^. Maintaining the model’s flexibility is essential for accurately capturing the data’s behaviour.

To ensure the validity of the distributional assumptions, we perform hypothesis tests for fixed effects and variance components, as well as employ diagnostic plots. The likelihood ratio test can assess hypotheses about two models with identical fixed effects and nested variance-covariance structures^5,32^. Moreover, we refit the models with the same variance-covariance structure using maximum likelihood estimation instead of restricted maximum likelihood estimation to test the fixed effects parameters. We then apply the Wald test to estimate the degrees of freedom via the Satterthwaite approximation^33^. Diagnostic plots we consider include a boxplot of residuals by group, standardised conditional residuals compared to fitted values, observed responses compared to fitted values within the group, and a normal probability plot of the conditional and marginal residuals with a 95% simulation envelope^34,35^.

To evaluate the predictive performance of our model and ensure that it can generalise to new data and make accurate predictions, we apply a comprehensive validation strategy that takes into account the hierarchical structure of the data. We split the data into two sets: a training set containing about 70–85% of the original data, which is used for parameter estimation and inference, and a test set containing the remaining 15–30%, which is used for confirmatory analysis to quantify how well the model performs in prediction. We propose two systematic validation approaches to assess the predictive performance of our multilevel model. The first approach is systematic validation by season and the second approach is validation using a game within a season.

For systematic validation by season, we perform the following steps: (1) divide the data into two groups, called the training set and the test set; (2) the test set consists of the *i*th level of the season, $i=1,2,\ldots ,I$, and the training set consists of the remaining $I-1$ seasons; (3) fit the model to the training set and evaluate against the test set; (4) summarise the prediction performance using the concordance correlation coefficient proposed by^36^, the Pearson correlation coefficient and the root mean square error (RMSE) between the true values and the predictions based on the test set; (5) repeat the process *I* times assuming that the probability that the test set consists of a season already used as a test is zero.

For validation using the game within season approach, we perform the following steps: (1) the data are divided into two groups, called the training set and the test set; (2) the test set consists of *I* subsets, where each subset is a random sample without replacement of 10% of the games played in the *i*th season, $i=1,2,\ldots ,I$, and each game has the same probability of being included in the sample; (3) the training data is then assembled from the remaining data; (4) repeat the process 100 times; (5) summarise the prediction performance using the average of the concordance correlation coefficient, the Pearson correlation coefficient and the root mean square error (RMSE) between the true values and the predictions based on 100 test sets.

It is worth noting that this approach allows us to make predictions that include a new level of season and possibly new levels of groups that are not included in the estimation model, such as a new athlete within a team or a new team. To make predictions, we use the unconditional (population level) to estimate the model, and we consider 1000 bootstrap samples of the model (3) to obtain confidence intervals for the prediction. However, predictions under a multilevel model can be more complicated than the classical regression approach^18^. Predictions for a new athlete at one of the pre-known levels of a team within an existing season are likely to be more accurate than predictions for a completely new season.

### The min–max scaling

The Min–Max scaling transformation is a common technique to normalise data and make it more interpretable. It rescales the data to a fixed range of values, usually between 0 and 1 or between a and b, while preserving the original relationships between the data points. In this case, we applied the Min–Max scaling to the athletes’ adjusted ON scores to make the scores more interpretable and easier to visualise.

The formula for the Min–Max scaling is given by:

6 $$\begin{aligned} \varvec{\gamma }^{*\tiny \text{(S) }} = a + \frac{\left( \varvec{\gamma }^{\tiny \text{(S) }} - \min {\left( \varvec{\gamma }^{\tiny \text{(S) }}\right) \left( b-a\right) }\right) }{\max {\left( \varvec{\gamma }^{\tiny \text{(S) }}\right) }-\min {\left( \varvec{\gamma }^{\tiny \text{(S) }}\right) }} \end{aligned}$$

where $\varvec{\gamma }^{*\tiny \text{(S) }}$ is the rescaled score, *a* and *b* are the lower and upper bounds of the range, and $\min {\left( \varvec{\gamma }^{\tiny \text{(S) }}\right) }$ and $\max {\left( \varvec{\gamma }^{\tiny \text{(S) }}\right) }$ are the minimum and maximum values of the original score.

In our case, we set $a=0$ and $b=100$ so that the rescaled scores can take values between 0 and 100. This range can be interpreted as a scale for the athletes’ performance in the game, with higher values indicating better performance. For example, if a particular athlete’s adjusted ON score decreases over the course of the match, this may indicate that the athlete’s performance is declining and the coach may consider replacing the athlete with another player.

It is important to note that while the Min–Max scaling may improve the interpretability of the results, it does not change the underlying statistical properties of the data. Therefore, any inferences or conclusions drawn from the rescaled scores should be based on the original statistical model and assumptions.

## Considerations and limitations in seasonal model weight updating for the ON score

A limitation of this study relates to updating model weights for ON scores at each season’s end, impacting their comparability across different seasons. This arises from the ON scores’ reliance on PCA weights, which reflect the variable covariance. Significant shifts due to outlier seasons or technological advancements could substantially alter these weights. Consequently, comparing ON scores over multiple seasons requires acknowledging potential discrepancies from weight changes. While re-calibrating past ON scores using updated weights is a potential remedy, its feasibility varies. This limitation presents a critical area for future research, particularly in longitudinal studies, where a comprehensive simulation study could effectively evaluate the implications of such re-calibrations.

To address the limitation regarding model weight updating for ON scores, practitioners can adopt a consistent and transparent approach. When updating weights at the end of a season, it is crucial to document the changes and consider their impact on score comparability. Practitioners could maintain a version history of weights used each season, allowing for historical ON score re-calibrations when necessary. Furthermore, it may be beneficial to perform sensitivity analyses to understand the extent of changes due to weight updates. This practice can provide insights into whether re-calibrations significantly alter the interpretation of an athlete’s performance across seasons. These steps will ensure that practitioners are equipped to handle the potential challenges of weight updating in longitudinal analyses.

### Supplementary Information

Supplementary Information 1.

Supplementary Information 2.

## Data Availability

The datasets generated and/or analysed during the current study are available in the Zenodo repository, https://doi.org/10.5281/zenodo.8056757.

## References

[1] Ortega E, Villarejo D, Palao JM (2009). Differences in game statistics between winning and losing rugby teams in the six nations tournament. J. Sports Sci. Med..

[2] Leite N, Baker J, Sampaio J (2009). Paths to expertise in Portuguese national team athletes. J. Sports Sci. Med..

[3] Hvattum LM (2019). A comprehensive review of plus-minus ratings for evaluating individual players in team sports. Int. J. Comput. Sci. Sport.

[4] Hass Z, Craig BA (2018). Exploring the potential of the plus/minus in NCAA women’s volleyball via the recovery of court presence information. J. Sports Anal..

[5] Baayen RH, Davidson DJ, Bates DM (2008). Mixed-effects modeling with crossed random effects for subjects and items. J. Mem. Lang..

[6] Matano, F., Richardson, L. F., Pospisil, T., Eubanks, C. & Qin, J. *Augmenting Adjusted Plus-Minus in Soccer with FIFA Ratings* 1–10 arXiv:1810.08032 (2018).

[7] Karipidis A, Fotinakis P, Taxildares K, Fatouros J (2001). Factors characterizing a successful performance in basketball. J. Hum. Mov. Stud..

[8] Lorenzo A, Gomez MA, Ortega E, Ibanez SJ, Sampaio J (2010). Game related statistics which discriminate between winning and losing under-16 male basketball games. J. Sports Sci. Med..

[9] Vilain, J.-B. & Kolkovsky, R. L. Estimating individual productivity in football. (2016).

[10] Gramacy RB, Jensen ST, Taddy M (2013). Estimating player contribution in hockey with regularized logistic regression. J. Quant. Anal. Sports.

[11] Macdonald, B. *Adjusted Plus-Minus for NHL Players using Ridge Regression with Goals, Shots, Fenwick, and Corsi* vol 8, 1–24, 10.1515/1559-0410.1447 (2012). arXiv:1201.0317.

[12] Franks AM, D’Amour A, Cervone D, Bornn L (2016). Meta-analytics: Tools for understanding the statistical properties of sports metrics. J. Quant. Anal. Sports.

[13] Deshpande SK, Jensen ST (2016). Estimating an NBA player’s impact on his team’s chances of winning. J. Quant. Anal. Sports.

[14] Rabaz FC, Castuera RJ, Arias AG, Domíguez AM, Arroyo MPM (2013). Relationship between performance in game actions and the match result. A study in volleyball training stages. J. Hum. Sport Exerc..

[15] Laird NM, Ware JH (1982). Random-effects models for longitudinal data. Biometrics.

[16] Guo G, Zhao H (2000). Multilevel modeling for binary data. Annu. Rev. Sociol..

[17] Fitzmaurice G, Davidian M, Verbeke G, Molenberghs G (2008). Longitudinal Data Analysis.

[18] Gelman A, Hill J (2006). Data Analysis Using Regression and Multilevel/Hierarchical Models.

[19] Pinheiro JC, Bates DM (2000). Mixed-Effects Models in S and S-PLUS.

[20] Deitch JR, Starkey C, Walters SL, Moseley JB (2006). Injury risk in professional basketball players: a comparison of women’s national basketball association and national basketball association athletes. Am. J. Sports Med..

[21] Murtagh F, Legendre P (2014). Ward’s hierarchical agglomerative clustering method: Which algorithms implement ward’s criterion?. J. Classif..

[22] Mettenheim HJV, Breitner MH (2014). Decision analytics with heatmap visualization for multi-step ensemble data. Bus. Inf. Syst. Eng..

[23] Rosenbaum, D. Measuring how NBA players help their teams win (2004).

[24] Kubatko J, Oliver D, Pelton K, Rosenbaum DT (2007). A starting point for analyzing basketball statistics. J. Quant. Anal. Sports.

[25] Ilardi, S. The next big thing: Real plus-minus (2014).

[26] Hollinger J (2004). Pro Basketball Forecast.

[27] Henderson, C. Estimation of genetic parameters. *Ann. Math. Stat.* 309–310. (1950).

[28] Henderson, C. Selection index and expected genetic advance. In *Statistical Genetics and Plant Breeding* 141–163 (National Academy of Sciences, National Research Council, 1963).

[29] Matuschek H, Kliegl R, Vasishth S, Baayen H, Bates D (2017). Balancing type I error and power in linear mixed models. J. Mem. Lang..

[30] West B, Welch KB, Galecki AT (2015). Linear Mixed Models: A Practical Guide Using Statistical Software.

[31] Woolson, R. F. Wilcoxon signed-rank test. *Wiley Encyclopedia of Clinical Trials* 7–9 (2008).

[32] Müller S, Scealy JL, Welsh AH (2013). Model selection in linear mixed models. Stat. Sci..

[33] Kuznetsova A, Brockhoff PB, Christensen RHB (2017). lmerTest package: Tests in linear mixed effects models. J. Stat. Softw..

[34] Nobre JS, Da Motta Singer J (2007). Residual analysis for linear mixed models. Biom. J..

[35] Moral RA, Hinde J, Demétrio CG (2017). Half-normal plots and overdispersed models in R: The hnp package. J. Stat. Softw..

[36] Lin LI (1989). A concordance correlation coefficient to evaluate reproducibility. Biometrics.

