# Supplementary material for: A hierarchical approach for evaluating athlete performance with an application in elite basketball
Source: Sci Rep. 2024 Jan 19;14:1717. doi: 10.1038/s41598-024-51232-2 (PMC10799012; doi:10.1038/s41598-024-51232-2)
Supplement: Supplementary file 1 — Supplementary Information 1. [file 41598_2024_51232_MOESM1_ESM.pdf]

THE ROSLIN INSTITUTE

A Hierarchical Approach for Evaluating Athlete Performance with an Application in Elite Basketball

Thiago de Paula Oliveira & John Newell

December 7, 2023

1 PARAMETER ESTIMATES

In this supplementary material, I am presenting the model parameter estimate, standard error, and t-test based on Satterthwaite's method. These statistical values are essential for understanding the results of the analysis and assessing the significance of the findings. The model parameter estimate represents the effect size of the independent variable on the dependent variable, while the standard error measures the degree of uncertainty or variability in the estimate. The t-test based on Satterthwaite's method is used to determine whether the estimate is significantly different from zero, which indicates whether the independent variable has a significant effect on the dependent variable. These values are crucial for interpreting the results of the analysis and making informed decisions based on the findings.

Table 1.1: Parameter estimates by REML, their standard error, and t-tests based on Satterthwaite's method

Source of Variation	Estimate	Std. Error	df	t	Pr(>|t|)
(Intercept)	1.0400	0.0026	3.1373	399.6312	<0.0001
VenueHome	-0.0008	0.0001	101802.2773	-10.6271	<0.0001
PositionF	0.0050	0.0021	10745.6127	2.3594	0.0183
PositionG	0.0063	0.0015	3631.8854	4.0495	0.0001
PositionPF	0.0050	0.0006	1972.9231	9.0277	<0.0001

Continued on next page

Table 1.1 – *Continued from previous page*

Source of Variation	Estimate	Std. Error	df	t	Pr(>|t|)
PositionPG	0.0083	0.0006	2045.7558	14.7303	<0.0001
PositionSF	0.0080	0.0006	1983.5182	14.1206	<0.0001
PositionSG	0.0098	0.0006	2000.4882	17.8548	<0.0001
RookieY	-0.0039	0.0005	2286.5441	-7.1874	<0.0001
poly(Pts, 3)1	17.9904	0.0449	103771.8579	400.9291	<0.0001
poly(Pts, 3)2	-3.1078	0.0428	102866.5354	-72.5803	<0.0001
poly(Pts, 3)3	1.5227	0.0398	102469.5079	38.2484	<0.0001
poly(BlkAgainst, 2)1	-5.7628	0.0314	102827.8883	-183.6957	<0.0001
poly(BlkAgainst, 2)2	-0.6031	0.0176	101920.7667	-34.2450	<0.0001
poly(Ast, 3)1	5.7431	0.0228	104030.6117	251.7640	<0.0001
poly(Ast, 3)2	-0.7072	0.0185	102960.0607	-38.3012	<0.0001
poly(Ast, 3)3	0.1354	0.0149	102228.5617	9.1005	<0.0001
poly(FtAtt, 2)1	4.5466	0.0859	102773.7422	52.9352	<0.0001
poly(FtAtt, 2)2	-0.4700	0.0462	102005.9199	-10.1654	<0.0001
poly(OffReb, 2)1	2.8015	0.0205	102993.1849	136.7166	<0.0001
poly(OffReb, 2)2	-0.1713	0.0196	102285.6988	-8.7593	<0.0001
PositionC:poly(Pts, 3)1	-0.3439	0.0626	100469.1637	-5.4915	<0.0001
PositionF:poly(Pts, 3)1	3.7910	1.4688	102440.0601	2.5810	0.0099
PositionG:poly(Pts, 3)1	1.7332	0.6409	102604.3068	2.7042	0.0068
PositionPF:poly(Pts, 3)1	-0.2848	0.0545	101417.2110	-5.2284	<0.0001
PositionPG:poly(Pts, 3)1	0.1579	0.0527	99223.0036	2.9955	0.0027
PositionSF:poly(Pts, 3)1	-0.1659	0.0536	98898.8469	-3.0930	0.0020
PositionC:poly(Pts, 3)2	0.2346	0.0539	103725.3993	4.3499	<0.0001
PositionF:poly(Pts, 3)2	4.8757	1.9734	102499.9207	2.4707	0.0135
PositionG:poly(Pts, 3)2	1.7238	0.9059	102234.7045	1.9029	0.0571
PositionPF:poly(Pts, 3)2	0.3108	0.0488	103586.5741	6.3712	<0.0001
PositionPG:poly(Pts, 3)2	-0.1070	0.0405	103940.5175	-2.6433	0.0082
PositionSF:poly(Pts, 3)2	0.1572	0.0429	103803.7625	3.6611	0.0003
PositionC:poly(Pts, 3)3	-0.1125	0.0512	102765.7209	-2.1964	0.0281
PositionF:poly(Pts, 3)3	2.3891	1.0590	102866.2656	2.2561	0.0241
PositionG:poly(Pts, 3)3	1.3777	0.5771	102539.9999	2.3875	0.0170
PositionPF:poly(Pts, 3)3	0.0008	0.0479	102747.8230	0.0172	0.9863
PositionPG:poly(Pts, 3)3	0.0308	0.0390	102974.2817	0.7913	0.4288
PositionSF:poly(Pts, 3)3	0.0901	0.0425	102977.9759	2.1195	0.0340
PositionC:poly(BlkAgainst, 1)	-0.4188	0.0468	102852.7015	-8.9576	<0.0001
PositionF:poly(BlkAgainst, 1)	-0.8118	0.1783	102774.0763	-4.5530	<0.0001
PositionG:poly(BlkAgainst, 1)	0.0667	0.1494	103888.2060	0.4469	0.6549
PositionPF:poly(BlkAgainst, 1)	-0.1991	0.0428	102749.1383	-4.6475	<0.0001
PositionPG:poly(BlkAgainst, 1)	0.0084	0.0416	102922.1834	0.2026	0.8395
PositionSF:poly(BlkAgainst, 1)	-0.0969	0.0431	102799.9785	-2.2470	0.0246
poly(Pts, 3)1:RookieY	0.4747	0.0891	103937.6016	5.3301	<0.0001

Continued on next page

Table 1.1 – *Continued from previous page*

Source of Variation	Estimate	Std. Error	df	t	Pr(>|t|)
poly(Pts, 3)2:RookieY	-0.0661	0.0993	102372.5057	-0.6659	0.5055
poly(Pts, 3)3:RookieY	0.5878	0.0895	102628.9203	6.5654	<0.0001
poly(BlkAgainst, 1):RookieY	0.2571	0.0488	102901.9936	5.2663	<0.0001
RookieN:poly(FtAtt, 1)	-0.4755	0.0726	103135.1161	-6.5525	<0.0001
poly(Pts, 3)1:poly(BlkAgainst, 2)1	528.8836	6.2420	102006.2130	84.7300	<0.0001
poly(Pts, 3)2:poly(BlkAgainst, 2)1	-237.2986	4.6629	101946.5934	-50.8902	<0.0001
poly(Pts, 3)3:poly(BlkAgainst, 2)1	108.4265	4.3432	101843.4269	24.9648	<0.0001
poly(Pts, 3)1:poly(BlkAgainst, 2)2	105.3237	5.1218	101943.4203	20.5638	<0.0001
poly(Pts, 3)2:poly(BlkAgainst, 2)2	-45.6901	4.0799	101918.8538	-11.1989	<0.0001
poly(Pts, 3)3:poly(BlkAgainst, 2)2	9.1870	3.0183	101846.3962	3.0438	0.0023
poly(Pts, 3)1:poly(Ast, 3)1	-558.5861	7.5188	102727.2803	-74.2919	<0.0001
poly(Pts, 3)2:poly(Ast, 3)1	243.6078	6.2220	102171.0665	39.1527	<0.0001
poly(Pts, 3)3:poly(Ast, 3)1	-124.9686	5.4605	102048.1057	-22.8861	<0.0001
poly(Pts, 3)1:poly(Ast, 3)2	153.3298	5.5771	102298.5754	27.4928	<0.0001
poly(Pts, 3)2:poly(Ast, 3)2	-98.1805	4.6936	102010.2525	-20.9180	<0.0001
poly(Pts, 3)3:poly(Ast, 3)2	74.4086	4.0258	102004.6047	18.4831	<0.0001
poly(Pts, 3)1:poly(Ast, 3)3	-42.1565	4.2492	101934.5951	-9.9211	<0.0001
poly(Pts, 3)2:poly(Ast, 3)3	32.9144	4.2328	101902.2804	7.7761	<0.0001
poly(Pts, 3)3:poly(Ast, 3)3	-39.0651	3.5934	101947.1215	-10.8713	<0.0001
poly(Pts, 3)1:poly(FtAtt, 3)1	-374.2573	13.9214	102009.4715	-26.8836	<0.0001
poly(Pts, 3)2:poly(FtAtt, 3)1	115.4141	9.5566	101888.2852	12.0769	<0.0001
poly(Pts, 3)3:poly(FtAtt, 3)1	-152.4784	6.8266	101841.7412	-22.3360	<0.0001
poly(Pts, 3)1:poly(FtAtt, 3)2	93.9055	11.1652	101915.7846	8.4105	<0.0001
poly(Pts, 3)2:poly(FtAtt, 3)2	-39.1598	6.2805	101877.3543	-6.2351	<0.0001
poly(Pts, 3)3:poly(FtAtt, 3)2	57.4484	3.4119	101898.1773	16.8379	<0.0001
poly(Pts, 3)1:poly(FtAtt, 3)3	-5.2567	4.0918	101753.0888	-1.2847	0.1989
poly(Pts, 3)2:poly(FtAtt, 3)3	-0.1788	3.4947	101738.4023	-0.0512	0.9592
poly(Pts, 3)3:poly(FtAtt, 3)3	-14.0180	1.7823	101870.2516	-7.8653	<0.0001
poly(Pts, 3)1:poly(OffReb, 2)1	-304.1799	7.0273	102471.5999	-43.2857	<0.0001
poly(Pts, 3)2:poly(OffReb, 2)1	148.3420	5.4072	102176.2716	27.4341	<0.0001
poly(Pts, 3)3:poly(OffReb, 2)1	-85.4357	4.7754	101987.6774	-17.8906	<0.0001
poly(Pts, 3)1:poly(OffReb, 2)2	41.6298	5.7634	102153.5540	7.2231	<0.0001
poly(Pts, 3)2:poly(OffReb, 2)2	-31.1912	4.4605	102071.0970	-6.9927	<0.0001
poly(Pts, 3)3:poly(OffReb, 2)2	32.8910	4.2113	101919.6996	7.8101	<0.0001
poly(BlkAgainst, 1):poly(Ast, 3)1	124.9668	4.6859	101994.6006	26.6688	<0.0001
poly(BlkAgainst, 1):poly(Ast, 3)2	-48.3387	3.7200	101907.9467	-12.9944	<0.0001
poly(BlkAgainst, 1):poly(Ast, 3)3	27.6642	3.7247	101785.6553	7.4273	<0.0001
poly(BlkAgainst, 1):poly(FtAtt, 3)1	67.0978	4.8453	101894.8108	13.8479	<0.0001
poly(BlkAgainst, 1):poly(FtAtt, 3)2	-28.3295	3.6552	101793.8044	-7.7505	<0.0001
poly(BlkAgainst, 1):poly(FtAtt, 3)3	10.6002	4.0719	101754.5013	2.6033	0.0092
poly(BlkAgainst, 1):poly(OffReb, 1)	49.8670	4.0743	102084.3192	12.2394	<0.0001

Continued on next page

Table 1.1 – *Continued from previous page*

Source of Variation	Estimate	Std. Error	df	t	Pr(>|t|)
poly(Ast, 1):poly(FtAtt, 2)1	-64.3199	5.1851	102360.1817	-12.4048	<0.0001
poly(Ast, 1):poly(FtAtt, 2)2	21.7036	4.0106	102013.1296	5.4115	<0.0001
poly(OffReb, 1):poly(Ast, 2)1	-49.4313	5.0026	102666.9268	-9.8812	<0.0001
poly(OffReb, 1):poly(Ast, 2)2	23.1757	4.3293	102167.7719	5.3532	<0.0001
poly(FtAtt, 1):poly(OffReb, 1)	-17.0715	4.5815	102158.1303	-3.7262	0.0002
