# Supplementary material for: A hierarchical approach for evaluating athlete performance with an application in elite basketball
Source: Sci Rep. 2024 Jan 19;14:1717. doi: 10.1038/s41598-024-51232-2 (PMC10799012; doi:10.1038/s41598-024-51232-2)
Supplement: Supplementary file 2 — Supplementary Information 2. [file 41598_2024_51232_MOESM2_ESM.pdf]

A Hierarchical Approach for Evaluating Athlete Performance with an Application in Elite Basketball

Thiago de Paula Oliveira & John Newell
June 19, 2023

Specifications of variance coefficient matrices

We consider the specifications of the general matrices under the model (3) for a given season i (V_i), or for a given team j within season i $\left(V_{ij}\right)$, or for a given athlete k within team j within season i $\left(V_{ijk}\right)$. First, we defined the variance of $\gamma^{(S)}_{ijkl}$, which is given by

$$\operatorname{Var}\left(\gamma^{(S)}_{ijkl}\right) = \sigma_1^2 + \sigma_2^2 + \sigma_3^2 + \sigma^2 = R_1,$$

and covariances of the joint variability of $\gamma^{(S)}_{ijkl}$ and $\gamma^{(S)}_{i'j'k'l'}$, with $ijkl \neq i'j'k'l'$, is given by

$$\operatorname{Cov}\left(\gamma^{(S)}_{ijkl}, \gamma^{(S)}_{ijkl'}\right) = \sigma_1^2 + \sigma_2^2 + \sigma_3^2 = R_2; \quad \operatorname{Cov}\left(\gamma^{(S)}_{ijkl}, \gamma^{(S)}_{ijk'l}\right) = \sigma_1^2 + \sigma_2^2 = R_3; \quad \operatorname{Cov}\left(\gamma^{(S)}_{ijkl}, \gamma^{(S)}_{ij'kl}\right) = \sigma_1^2;$$

$$\operatorname{Cov}\left(\gamma^{(S)}_{ijkl}, \gamma^{(S)}_{i'jkl}\right) = 0; \quad \operatorname{Cov}\left(\gamma^{(S)}_{ijkl}, \gamma^{(S)}_{ijk'l'}\right) = \sigma_1^2 + \sigma_2^2 = R_3; \quad \operatorname{Cov}\left(\gamma^{(S)}_{ijkl}, \gamma^{(S)}_{ij'kl'}\right) = \sigma_1^2;$$

$$\operatorname{Cov}\left(\gamma^{(S)}_{ijkl}, \gamma^{(S)}_{i'jkl'}\right) = 0; \quad \operatorname{Cov}\left(\gamma^{(S)}_{ijkl}, \gamma^{(S)}_{ij'k'l}\right) = \sigma_1^2; \quad \operatorname{Cov}\left(\gamma^{(S)}_{ijkl}, \gamma^{(S)}_{i'jk'l}\right) = 0;$$

$$\operatorname{Cov}\left(\gamma^{(S)}_{ijkl}, \gamma^{(S)}_{i'j'kl}\right) = 0; \quad \operatorname{Cov}\left(\gamma^{(S)}_{ijkl}, \gamma^{(S)}_{ij'k'l'}\right) = \sigma_1^2; \quad \operatorname{Cov}\left(\gamma^{(S)}_{ijkl}, \gamma^{(S)}_{ij'k'l'}\right) = \sigma_1^2;$$

$$\operatorname{Cov}\left(\gamma^{(S)}_{ijkl}, \gamma^{(S)}_{i'j'kl'}\right) = 0; \quad \operatorname{Cov}\left(\gamma^{(S)}_{ijkl}, \gamma^{(S)}_{i'jk'l'}\right) = 0; \quad \operatorname{Cov}\left(\gamma^{(S)}_{ijkl}, \gamma^{(S)}_{i'j'k'l}\right) = 0;$$

Based on the variance and covariances defined above, the variance-covariance matrices can be defined as follows

$$\mathbf{V}_{ijk} = \begin{bmatrix} R_1 & R_2 & \dots & R_2 \\ R_2 & R_1 & \dots & R_2 \\ \vdots & \vdots & \ddots & \vdots \\ R_2 & R_2 & \dots & R_1 \end{bmatrix}; \quad \mathbf{V}_{ij} = \begin{bmatrix} \mathbf{V}_{ijk} & \mathbf{R}_3 & \dots & \mathbf{R}_3 \\ \mathbf{R}_3 & \mathbf{V}_{ijk} & \dots & \mathbf{R}_3 \\ \vdots & \vdots & \ddots & \vdots \\ \mathbf{R}_3 & \mathbf{R}_3 & \dots & \mathbf{V}_{ijk} \end{bmatrix}; \quad \mathbf{V}_i = \begin{bmatrix} \mathbf{V}_{ij} & \mathbf{R}_4 & \dots & \mathbf{R}_4 \\ \mathbf{R}_3 & \mathbf{V}_{ij} & \dots & \mathbf{R}_4 \\ \vdots & \vdots & \ddots & \vdots \\ \mathbf{R}_4 & \mathbf{R}_4 & \dots & \mathbf{V}_{ij} \end{bmatrix},$$

where

$$\mathbf{R}_3 = \begin{bmatrix} R_3 & R_3 & \dots & R_3 \\ R_3 & R_3 & \dots & R_3 \\ \vdots & \vdots & \ddots & \vdots \\ R_3 & R_3 & \dots & R_3 \end{bmatrix}; \text{ and } \mathbf{R}_4 = \begin{bmatrix} \sigma_1^2 & \sigma_1^2 & \dots & \sigma_1^2 \\ \sigma_1^2 & \sigma_1^2 & \dots & \sigma_1^2 \\ \vdots & \vdots & \ddots & \vdots \\ \sigma_1^2 & \sigma_1^2 & \dots & \sigma_1^2 \end{bmatrix}.$$
